# Sampling via the aggregation value for data-driven manufacturing

**DOI:** 10.1093/nsr/nwac201

**Published:** 2022-09-24

**Authors:** Xu Liu, Gengxiang Chen, Yingguang Li, Lu Chen, Qinglu Meng, Charyar Mehdi-Souzani

**Affiliations:** School of Mechanical and Power Engineering, Nanjing Tech University, Nanjing 211816, China; College of Mechanical & Electrical Engineering, Nanjing University of Aeronautics and Astronautics, Nanjing 210016, China; College of Mechanical & Electrical Engineering, Nanjing University of Aeronautics and Astronautics, Nanjing 210016, China; College of Mechanical & Electrical Engineering, Nanjing University of Aeronautics and Astronautics, Nanjing 210016, China; College of Mechanical & Electrical Engineering, Nanjing University of Aeronautics and Astronautics, Nanjing 210016, China; University Research Laboratory in Automated Production, École normale supérieure Paris-Saclay, Université Paris-Saclay, Université Sorbonne Paris Nord, Gif-Sur-Yvette 91190, France

**Keywords:** data-driven modelling, intelligent manufacturing, data sampling, data value

## Abstract

Data-driven modelling has shown promising potential in many industrial applications, while the expensive and time-consuming labelling of experimental and simulation data restricts its further development. Preparing a more informative but smaller dataset to reduce labelling efforts has been a vital research problem. Although existing techniques can assess the value of individual data samples, how to represent the value of a sample set remains an open problem. In this research, the aggregation value is defined using a novel representation for the value of a sample set by modelling the invisible redundant information as the overlaps of neighbouring values. The sampling problem is hence converted to the maximisation of the submodular function over the aggregation value. The comprehensive analysis of several manufacturing datasets demonstrates that the proposed method can provide sample sets with superior and stable performance compared with state-of-the-art methods. The research outcome also indicates its appealing potential to reduce labelling efforts for more data-scarcity scenarios.

## Teaser

A new concept, aggregation value, is proposed to evaluate the real value of sample set, thus high-value sampling can be ensured which will greatly reduce the labelling effort for machine learning tasks.

## INTRODUCTION

With the trend of rapid digitisation and intelligentisation in the manufacturing industry, process modelling has become the fundamental technology for extracting industrial knowledge and revealing hidden laws [1]. Many non-linear multi-physics dynamics accompanying manufacturing processes bring significant difficulties in traditional mechanism-based modelling [2]. With the substantial development of multi-sensors and machine learning techniques, data-driven modelling, which can build a low-demand end-to-end solution for domain knowledge, has shown promising potential in diagnostics, decision-making and many other aspects of manufacturing [3]. However, the significant performance of data-driven modelling heavily relies on a large amount of labelled data for training, while generating labelled data in manufacturing is usually expensive and time-consuming either computationally or experimentally. For example, it would normally take several hours or days to conduct the complete three-dimensional (3D) thermo-chemical analysis of a typical aerospace composite part (e.g. the wing skin of the Boeing 787) using commercial simulation software [4–6]. Consequently, the high computational cost limits the application of data-driven thermo-chemical models. Therefore, establishing data-driven models using substantially reduced labelled data is one of the most challenging tasks for intelligent manufacturing [7].

Previous research reported that, by leveraging auxiliary rich labelled data from one or multiple relevant tasks, supervised transfer learning [7], few-shot learning [8] or meta-learning [9] can enhance the performance of the target task when only a few labelled data are available. Furthermore, integrating physical knowledge can also reduce the data requirement [10,11]. A series of physics-informed or theory-guided machine learning methods are developed for different manufacturing scenarios, including milling stability analysis [12], composite curing [13] and tool wear monitoring [14]. To tackle the challenge of data scarcity, another essential issue emerged of how to determine the distribution of the limited labelled dataset. Since the distribution of the training data influences the performance of algorithms, sampling an informative data set that preserves the characteristics of the task can significantly reduce the required amount of training data [15–17].

Representativeness is the most common consideration for unsupervised sample selection problems, where the selected samples are expected to represent the characteristics that should be preserved [17]. Clustering sampling or probabilistic sampling methods can provide a reasonable sample set to approximate the probabilistic distribution of the potential total dataset [18,19]. Low-rank-based methods can select the fewest samples to preserve the patterns or basis for high-dimensional samples [15,20]. An important underlying presupposition of representativeness-based sampling methods is that we believe representative samples might provide more valuable information for the model [16]. Although reasonable, the presupposition is insufficient because representativeness is only the indirect characterisation of the value of samples. Thus, some core samples that can reflect the characteristic of the models might not be captured. This problem is further exacerbated in highly imbalanced real-world datasets where the representative sample set, with high probability, may miss the dominant samples [19].

To directly quantify the contribution of each sample during the model training, recent researchers proposed another interesting indicator, the value of samples. The value function $v$(*x*) is then defined as the function that can quantitatively measure the value of the sample *x* with respect to a given learning algorithm and a performance metric [21]. The first attempt at data valuation was leave one out and the subsequent influence function method [22], in which a specific value was determined for each sample according to the performance difference when the sample was removed from the data pool. A fundamental limitation is that these methods can only represent the marginal gain for one specific sample set N∖{*i*}. From the perspective of cooperative game theory, training a dataset can be treated as a coalitional game, in which all data samples are players working for a common goal. Based on cost-sharing theory, Ghorbani *et al.* [21] revolutionarily introduced Shapley value into data valuation and sample selection problems. The Shapley value of each sample was represented as the average marginal gains of all potential subsets. Then highest-ranking samples were selected as the satisfied sample set based on the standard greedy algorithm in submodular function maximisation [23,24]. A series of improved versions and accelerating algorithms were further proposed to boost the development of the Shapley value in the machine learning field [21].

Although the Shapley value can provide a ‘favourable and fair’ valuation of data, we found that the highest value sample set sometimes reduced the data diversity, especially when the size of the sample set was small, which would lead to a high generalisation error [25]. Further analysis in multiple datasets revealed the high-value samples' clustering phenomenon in feature space. Neighbouring samples from the same high-value cluster might carry similar or redundant feature information, which cannot bring a proportional contribution to the model training. Therefore, close or similar samples can only provide very small additional contributions for machine learning tasks, regardless of regression, classification or structural learning tasks [26,27]. This means that the sum of the values of samples in the selected set cannot represent the actual value of the set. Therefore, defining the actual value of a sample set considering redundant information becomes the critical challenge for sample selection problems.

In this paper, a novel ‘aggregation value’ is defined to represent the actual value of a sample set by explicitly aggregating the values of neighbouring samples; the optimal sample set can be obtained by greedily maximising a submodular function. Comprehensive experiments on several manufacturing datasets demonstrate the influence of data distribution on model performance and the enormous potential of data sampling. On the one hand, the selected optimised samples can provide more accurate and robust prediction results under the exact size of labelled data. On the other hand, the size of labelled data can be reduced by more than 50% under the fixed accuracy requirements. In addition, detailed analysis reveals the high-value samples' clustering phenomenon and interprets the advancement of the proposed aggregation-value-based sampling.

## AGGREGATION-VALUE-BASED SAMPLING

**Figure 1.**
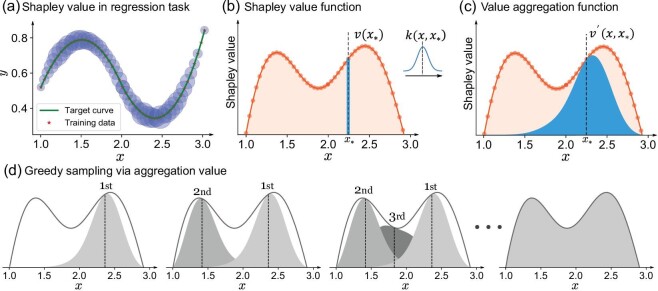
Illustration of the aggregation-value-based sampling. (a) Shapley value in a regression task; the sizes of the circles represent the Shapley values of each point. A larger circle means a more valuable sample. (b) Shapley value function. (c) Value aggregation function, representing the neighbouring influence of the value function. (d) Aggregation-value-based sampling via greedy maximisation.

The general illustration of the proposed aggregation-value-based sampling method is given in Fig. 1. Consider a curve regression task *y* = *f*(*x*) with 50 samples *x* ∈ [1, 3]. The Shapley values of these samples can be evaluated based on game theory to represent their average contribution during curve regression (Fig. 1a). A value function $v$(*x*) can then be established to assess the Shapley value of any potential samples (Fig. 1b).

To represent the values of neighbouring samples, a value aggregation function (VAF) is constructed by aggregating the values of its neighbouring samples using a kernel filter (Fig. 1c). Therefore, the close samples would share significant overlaps in their VAFs, which can explicitly represent the redundant information carried by these samples. Since the close high-value samples cannot increase the ‘area’ of VAFs, the union of individual VAFs becomes a reasonable representation of the actual value of a sample set. Based on this, the aggregation value, defined as the expectation of the united VAFs, can be the intuitive target to assess the sampling results. Maximising the aggregation value can effectively reveal the most contributing samples while mitigating redundant information. Figure 1d shows the procedure of greedy sampling, which queries the new potential sample by iteratively maximising the increment of the aggregation value.

**Figure 2.**
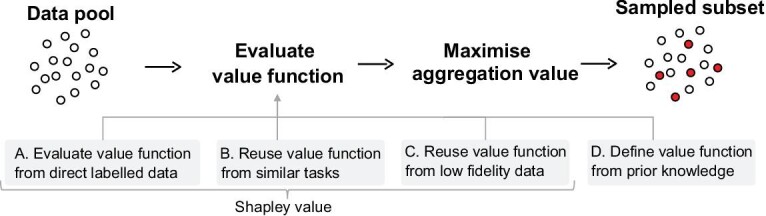
The implementation procedure of the proposed aggregation-value-based sampling method. The value function can be evaluated from four schemes: evaluate the value function from direct labelled data; reuse the value function from similar tasks; reuse the value function from low-fidelity data; define the value function from prior knowledge.

The implementation procedure of the proposed aggregation-value-based sampling is shown in Fig. 2, where a value function for the data pool is established first, followed by maximising the aggregation value for sampling. The purpose of aggregation-value-based sampling is to reduce the labelling efforts for industrial applications by designing an optimal but smaller sample set; thus, it is less meaningful if the establishment of the value function requires too much labelled data. Although the proposed method is derived from the Shapley value function, the basic idea of the aggregation value can be generalised to other forms of the value function as long as it is positively correlated with the real contribution of samples. Therefore, we generalise the proposed method to more practical scenarios in the case studies by introducing four value function schemes.

- **Scheme A: evaluate the value function from direct labelled data.** Sufficient direct labelled data could provide a more accurate value function but increase the labelling burden. Therefore, the case study for this scheme aims to demonstrate that the proposed method could find the optimal sample set rather than focusing on comparing the labelling efforts.
- **Scheme B: reuse the value function from similar tasks.** Just as transfer learning and meta-learning can utilise data from similar or relevant tasks to assist the target task, the value function from similar tasks, such as different manufacturing systems or cutting conditions, could also provide a reference for the target task.
- **Scheme C: reuse the value function from low-fidelity data**. High-fidelity manufacturing process simulation is expensive and time-consuming, while simplified low-fidelity models are far more efficient. Although not accurate enough, the low-fidelity data can still provide an effective value function to design the optimal samples for the following high-fidelity simulations.
- **Scheme D: define the value function from prior knowledge**. With a broad and in-depth understanding of the prior knowledge of various manufacturing processes, researchers and engineers can define specific value functions according to the sample requirements. From this point of view, aggregation-value-based sampling can be extended to various engineering-based sampling scenarios, such as curvature-based sampling for surface measurement[28] and adaptive sampling for aerodynamic modelling[29].

In the following section we report the case studies for the four schemes. Scheme A demonstrates that aggregation-value-based sampling could find the optimal sample sets for various engineering problems, including classification and regression. Schemes B, C and D show that the proposed method can reduce the labelling efforts while achieving similar prediction accuracy. The sensitivity analysis in the Results section shows that aggregation-value-based sampling is robust to the accuracy of value functions. This property provides the guarantee for reusability of value functions.

## RESULTS

### Scheme A: evaluate the value function from direct labelled data

**Figure 3.**
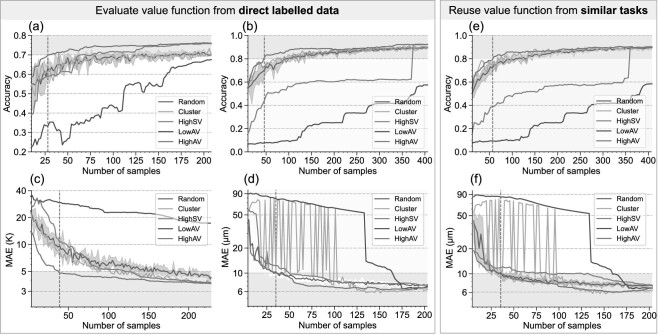
Comparison of different sampling methods. The value functions in (a–d) are evaluated from direct labelled data. The value functions in (e and f) are reused from similar tasks. (a) Experimental results of Cifar10. (b) Experimental results of CWRU HP1. (c) Experimental results of thermal lag prediction of the composite. (d) Experimental results of Tool wear B3C6. (e) Results of reusing the value function of task CWRU HP0 on HP1. (f) Results of reusing the value function of task B2C4 on B3C6.

Figure 3a–d reports the detailed results of different sampling methods on four datasets. A different number of samples is selected from the potential data pool. A machine learning model is then trained on the selected samples and evaluated on the test set. ‘HighAV’ (high aggregation value) is the high valuable dataset sampled by the proposed aggregation-value-based sampling method. ‘HighSV’ (high Shapley value) means sampling the high-Shapley-value samples greedily to construct the sample set [21]. ‘Cluster’ is the clustering-based core set selection strategy [19]. Lastly, ‘Random’ means generating the sample set randomly. The detailed data processing and model training are reported in the online supplementary material (S2, S3). A brief description of these tasks is summarised as follow.

(1) **Image classification.** Cifar10 [30] is a widely used classification dataset in the image process field. A small dataset is constructed from Cifar10 to evaluate the generalisability of the proposed method, and the result is shown in Fig. 3a.
(2) **Rolling bearing fault classification.** The bearing fault dataset from Case Western Reserve University (CWRU) [31] is a famous benchmark dataset in the fault diagnosis field. The vibration signals of normal and faulty conditions are collected under different motor loads (HP = 0,1,2,3). Two 10-way classification problems (HP = 0, HP = 1) are formulated, and the classification accuracies with varying numbers of samples from all methods are shown in Fig. 3b (HP = 1).
(3) **Composite curing.** Predicting the thermal lag of temperature from curing parameters is an important task for the quality control of composite parts. Six hundred combinations of curing parameters are generated from a reasonable range, and the corresponding thermal lags are simulated using finite element (FE) software [5,6]. The mean absolute error (MAE) results are shown in Fig. 3c.
(4) **Tool wear prediction.** The tool wear dataset from the Prognostics and Health Management Society [32] consists of collected monitoring signals during milling and the corresponding tool wear values for three blades of cutting tools. Two regression tasks are formulated: blade No. 2 of cutting tool No. 4 and blade No. 3 of cutting tool No. 6, denoted B2C4 and B3C6 for short. The MAE results are shown in Fig. 3d (B3C6).

Figure 3a and d illustrates that HighAV can consistently achieve superior performance, especially when the number is limited. Under most circumstances, HighAV outperforms the uncertainty boundary of Random (grey region) while Cluster is only better than Random occasionally but far more unstable. The Cluster results fluctuate sharply because similar sample sizes (e.g. 49 and 51 in Fig. 3d) can lead to totally different clusters, and some high-value samples might be missed. In Fig. 3b, the results of HighSV show ‘step effect’, namely, suddenly increasing at some point (around 350 in Fig. 3b). Theoretically, minimising the aggregation value can also provide the worst sample set. As seen in Fig. 3, ‘LowAV’ (low aggregation value), sampled by greedily minimising the aggregation value, can always provide far worse results than the lowest bound of Random. Although the low valuable sample set seems meaningless for real application, it does reveal the importance of the distribution of training data, as well as the magic of aggregation-value-based sampling.

Table 1 summaries the regression and classification results with training data from different sampling methods under different sample sizes (30, 50, 80, 100). It is clear that the proposed aggregation-value-based sampling method can provide better sample sets compared to other sampling methods.

**Table 1.** Summary of performance with training data from different sampling methods (Random, Cluster, HighSV, HighAV). The best results are highlighted bold. This table shows that HighAV can achieve better performance under the same number of samples.

	CWRU HP1 (ACC/%)		Cifar10 (ACC/%)		Tool B3C6 (MAE/μm)		Composite (MAE/K)	
Sample size	50	100	30	80	50	100	30	80
Random	73.18	80.52	61.47	69.60	9.32	7.98	10.90	6.54
Cluster	74.80	81.56	62.62	66.56	72.20	8.16	9.90	5.95
HighSV	48.57	55.84	59.00	70.62	9.90	7.98	16.54	5.87
HighAV	**83.12**	**87.79**	**70.20**	**74.71**	**8.88**	**6.70**	**5.70**	**4.41**

ACC means the accuracy of classification.

### Scheme B: reuse the value function from similar tasks

To avoid data labelling for the value function, in this section we investigate the possibility of reusing the value function learnt from a similar task on the target task without training a new one. Fig. 3e reports the cross-task application of reusing the value function from CWRU HP0 on HP1. It can be observed that the accuracy of HighSV is even lower than Random, but HighAV can consistently achieve leading performance. This phenomenon reveals that the effectiveness of HighSV relies heavily on the accuracy of the value function. However, HighAV is more robust, meaning that a less accurate value function can still provide helpful value information. The same conclusions can also be drawn from Fig. 3f, which reports results of the cross-task application of reusing the value function of B2C4 on B3C6.

### Scheme C: reuse the value function from low-fidelity data

In this section we investigate Scheme C for the composites curing case, in which the value function is first calculated from the simplified low-fidelity finite difference (FD) model, and then reused for parameters designed in high-fidelity FEM simulations.

**Figure 4.**
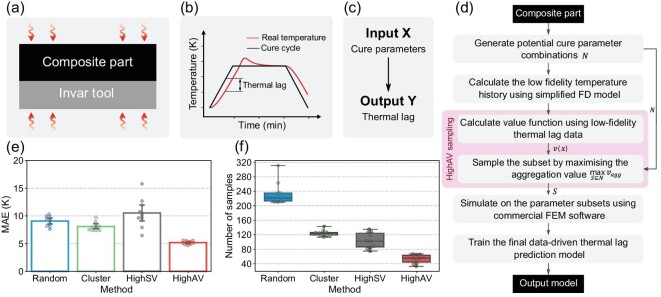
Experimental results of Scheme C, the thermo-chemical analysis of the composite. (a) Illustration of the 1D composite-tool curing system. (b) The cure cycle and the thermal lag in composite curing. (c) The defined data-driven task from the curing parameters to the corresponding thermal lag. (d) The full workflow of sampling curing parameters for composite simulation. (e) MAEs of 10 repeated trails of different sample selection methods with 40 samples. (f) Required samples of different sample selection methods to achieve an MAE of 5 K.

An illustration of the curing of a 1D composite-tool system is shown in Fig. 4a. The actual temperature of the composite part always lags behind the designed cure cycle (Fig. 4b). Thus, the thermal lag is defined as the maximum difference between the cure cycle and the actual temperature of any point in the thickness during the heat-up step [5,6]. The objective here is to establish the data-driven prediction model of thermal lag from the simulation results, where the input features include the heating rate, the cooling rate, the hold temperature, the hold time and the heat transfer coefficients of both sides (Fig. 4c). Since the labelled data comes from the time-consuming high-fidelity FEM simulation, a better sampling method should reduce the number of simulations but maintain the required accuracy of the data-driven model.

The detailed procedure of aggregation-value-based sampling for this case is shown in Fig. 4d. Six hundred potential curing parameters are generated first, and the corresponding thermal lags are calculated using a simplified FD model for obtaining the value function $v$(*x*). An optimal parameter sample set *S* is then determined based on the proposed sampling method for the subsequent complete high-fidelity FEM simulations.

To compare the influence of the data sampling on the performance of the data-driven model, a parameter sample set with *n* = 40 instances is selected from the potential data pool by different sampling methods. A Gaussian process regression model is then trained on the simulation results of the selected samples and evaluated on the test set. The MAEs of 10 repeated trials for four methods are shown in Fig. 4e. It can be observed that HighAV can achieve a superior and stable performance with an MAE around 5 K. Conversely, Cluster is slightly better than Random, and HighSV is very unstable, even worse than Random. These results show that the distribution of the designed curing parameter combinations significantly influences the performance of data-driven models, and the proposed HighAV can provide a better sample set stably. Figure 4f reports how many samples are required to achieve an MAE of 5 K for different sample selection methods. In each independent experiment, a sample set is constructed by increasing instances one by one from an empty set until the MAE becomes less than 5 K stably. The size of the final sample set is recorded as the required size of this trial. As shown in the scatter and box plots of 10 repeated tests in Fig. 4f, HighAV can achieve an MAE of 5 K with around 50 samples, much less than the required number for Random and Cluster. Table 2 reports the detailed required samples for different sampling methods to stably achieve MAEs of 5 and 6 K. These results demonstrate that the proposed sampling method can reduce the data-collecting effort of FEM simulations in the composite curing problem while maintaining the required accuracy.

**Table 2.** The required number of samples M for different sampling methods to achieve a predefined required MAE. Considering the uncertainties of different methods, the number M is defined as follows: during the sampling from 20 to 520 points, for any sample set with more than M samples, the MAE is always less than the required one. Here A±B represents the mean (A) and standard deviation (B) of the required number M in 10 repeated trials.

	Composite		Surface	
MAE	6 K	5 K	0.1 mm	0.01 mm
Random	172 ± 21	234 ± 30	122 ± 10	292 ± 8
Cluster	83 ± 5	124 ± 8	85 ± 3	199 ± 4
HighSV	68 ± 8	104 ± 21	331 ± 8	–
HighAV	25 ± 5	53 ± 11	62 ± 2	147 ± 1

### Scheme D: define the value function from prior knowledge

Dimensional inspection and reconstruction of engineering products comprising free-form surfaces requires accurate measurement of a large number of discrete points using a coordinate measuring machine with a touch-trigger probe [33]. An efficient sampling method should enable the reconstruction of the surface under the required accuracy with a limited amount of measurement points. Curvature and other geometric features are widely used prior knowledge for traditional measurement sampling methods. By defining the curvature of the surface as the value function $v$(*x*), the proposed aggregation-value-based sampling method can also be extended to sample the measurement points for the further surface reconstruction.

**Figure 5.**
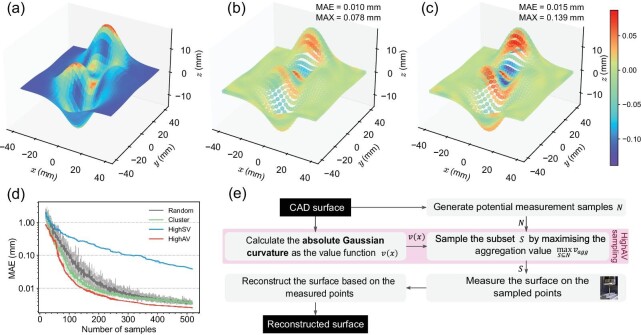
Experimental results of the surface measurement and reconstruction, defining the value function from the absolute Gaussian curvature. (a) The absolute Gaussian curvature function of peaks surface. (b) The error map of the surface reconstructed from the 140 points sampled by HighAV. The mean absolute and the maximum errors are 0.010 and 0.078 mm, respectively. (c) The error map of the surface reconstructed from the 140 points sampled by Cluster. The mean absolute and the maximum errors are 0.015 and 0.139 mm, respectively. (d) The relationship between the number of samples and the MAE of the reconstructed surfaces for different sampling methods. (e) The full workflow of sampling measurement points for the surface measurement and reconstruction.

The simulated measurements and reconstructed results of a matlab^®^ peak surface are shown in Fig. 5. The absolute Gaussian curvature function in Fig. 5a is defined as the value function for the subsequent sampling. Figure 5b is the error distribution map of the reconstructed surface with 140 measurement points sampled by HighAV. The MAE is 0.010 mm and the maximum absolute error (marked as MAX) is 0.078 mm. Figure 5c shows the errors of the surface reconstructed with 140 points sampled by Cluster. MAE and MAX are 0.015 and 0.139 mm, respectively. It is clear that HighAV can reduce the error of areas with high curvatures, which plays a similar role as traditional curvature-based sampling. Figure 5d reports the MAEs of four sampling methods with different numbers of samples ranging from 20 to 520. HighAV has a small MAE for almost any size of sample. Table 2 reports the required samples for different sampling methods to stably achieve MAEs of 0.1 and 0.01 mm. It is clear that HighAV can reduce the required measurement points under the predefined MAE. The full workflow of sampling measurement points is shown in Fig. 5e and the detailed experimental settings are reported in the online supplementary material (S2.5).

### Characteristics analysis

The abovementioned results show that aggregation-value-based sampling can provide superior and stable sample sets compared with Shapley-value-based or representativeness-based methods. This section comprehensively analyses the characteristics of aggregation-value-based sampling on the composite task and explains why it works. The analyses of other cases are reported in the online supplementary material (S4).

**Figure 6.**
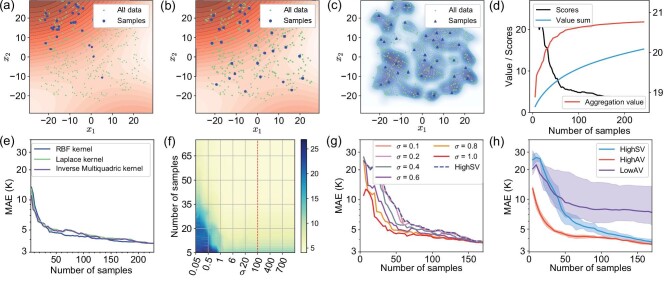
Characteristics analysis of the composites task. (a) A sample set of the composite task generated by HighSV. The contour map represents the Shapley value field, and the darker colour represents a larger value. (b) A sample set of the composite task generated by HighAV. (c) A sample set of the composite task generated by Cluster. The contour map is the kernel density estimation result of the samples’ distribution in the dataset. (d) The function between the number of samples and the corresponding MAE. (e) The sensitivity of HighAV on different kernel functions. (f) The sensitivity of HighAV on the parameter σ. (g) The degeneration from HighAV to HighSV of the composite task with different σ. (h) The sensitivity of HighAV on the random error of the Shapley value on the composite task.

Figure 6a–c shows the *t*-distributed stochastic neighbour embedding visualised features of samples in the composite task. These samples are generated by HighSV, HighAV and Cluster. The sampled points are marked with large points, and all points of the dataset are marked with small points.

The contour map in Fig. 6a and b represents the Shapley value field, and the darker of the contour map, the larger the Shapley value). Almost all the samples in Fig. 6a are concentrated in the upper left high-value area, showing that the high-value samples' clustering phenomenon would result in more similar and closed samples. Shapley-value-based sampling tends to be deficient because the sample set does not represent the dataset. More experimental results about the high-value samples' clustering phenomenon are provided in the online supplementary material (S4.1).

The contour map in Fig. 6c is the kernel density estimation result of the samples’ distribution in the dataset. The sample set of Cluster is representative of the probabilistic density of the dataset. Still, samples in the high-value area are random and insufficient, which could result in the unstable fluctuation of Cluster, as in Fig. 3d. As shown in Fig. 6b, the sample set generated by HighAV takes both the Shapley value and probabilistic density into consideration and provides a balanced and reasonable result.

Because of the redundant information, the functions between the number of samples and the corresponding performance of the data-driven model are usually approximately logarithmic (curve of Scores in Fig. 6d). Therefore, the direct sum of the Shapley value of all samples (marked as `Value sum' in Fig. 6d) cannot reasonably reflect the variation trend of the performance. However, the aggregation value curve in Fig. 6d can provide a more correlative evaluation of the actual performance.

### Sensitivity analysis

In this section we analyse the sensitivity of the proposed method on the composite task. As shown in Fig. 1c, the kernel function plays an important role in aggregating the neighbouring values and constructing VAFs. Figure 6e compares the HighAV results for the composite case with different kernel functions: the radial basis function (RBF) kernel, Laplace kernel and inverse multiquadric kernel. It is clear that different kernel functions have comparable and similar MAE convergence curves despite slight differences. We select RBF kernel *k*(*x*, x_*_) = exp ( − ‖*x* − x_*_‖^2^/σ) for all case studies because it is simple and general enough. The bandwidth parameter of the kernel function σ, which determines the influence range, is the one and the only parameter in the aggregation-value-based sampling. When σ is too small, the neighbouring values will not be aggregated, and aggregation-value-based sampling will degenerate into Shapley-value-based sampling. On the other hand, aggregation-value-based sampling will be less effective when σ is too large, because the aggregation value of all samples could be too similar to be distinguished.

Figure 6f shows performance on the composite task concerning σ from 0.05 to 1000. The darker shade means worse performance, and the dotted line is the selected parameter in the previous experiments. The large light yellow region implies that the aggregation-value-based sampling can achieve relatively robust performance for a wide range of σ and the performance is almost consistent for σ ∈ [1, 1000].

Figure 6g shows the degeneration process of the method as σ becomes smaller. HighAV turns to HighSV gradually from σ = 1 to σ = 0.1. The variations of MAE can also be observed in the bottom left corner of Fig. 6g.

Since the calculation of the Shapley value always brings random errors, we also analyse the sensitivities of HighSV, HighAV and LowAV with five random trials of the Shapley value function. As shown in Fig. 6h, HighAV is far more stable and robust than HighSV. For HighSV, the slight random error of the Shapley value changes the samples significantly, thus reducing the stability and robustness. However, aggregation-value-based sampling can aggregate the values of neighbouring samples by a kernel function, which plays the role of a smoothing filter, so that HighAV can be less sensitive to the random error of the Shapley value. The robustness of the proposed method enables the value function reuse and prior-knowledge-based value function in Schemes B, C and D.

## METHODS

### Shapley value

The value of a single data point in a potential data pool can be interpreted as how much improvement it can bring to the performance of the model. Suppose that there are *n* data points in the potential data set *N* and an indicator function $\varphi : 2^{|N|} \rightarrow \mathbb {R}$ that can represent the performance of any subset of the data pool. For a subset *S*⊆*N* and a given learning algorithm $\mathcal {A}$, the indicator function $\varphi (S, \mathcal {A})$ can either be the accurate rate in a classification problem or the MAE in a regression problem. The Shapley value of data point x_*_ ∈ *N* can be given as the average marginal gain for all potential subsets [21]:

(1) \begin{equation*} v(x_{*})=\frac{1}{n!} \sum _{S \subseteq N \backslash \lbrace x_{*}\rbrace } \frac{\varphi (S \cup \lbrace x_{*}\rbrace )-\varphi (S)}{{n-1\choose |S|}}. \end{equation*}

After obtaining the Shapley values of all samples in an initially labelled dataset, a general Shapley value function $v$(*x*) can be learnt from the paired data [{*x*_1_, $v$(*x*_1_)}, {*x*_2_, $v$(*x*_2_)}, …, {*x*_*n*_, $v$(*x*_*n*_)}] to predict the value of samples out of the dataset. A detailed computation of the Shapley value is given in the online supplementary material (S1.2).

### Sampling via aggregation value

#### Value aggregation considering neighbouring influences

For a machine learning task, it is more reasonable to evaluate the contribution of one sample considering the value of its neighbourhoods rather than only its own value. Suppose that the aggregation coefficients, which describe the weights when aggregating the value of neighbouring samples, are positively correlated with the Euclidean distance between samples in the feature space; then the VAF of given instance x_*_ is designed by adding a kernel filter to the value function as

(2) \begin{equation*} v^{\prime }(x, x_{*})=v(x) k(x, x_{*}). \end{equation*}

The kernel function *k*(*x*, x_*_) can influence how much information is aggregated from neighbouring samples. The performance comparisons of different kernel functions are reported in Fig. 6e. VAF $v$^′^(*x*, x_*_) can express the neighbouring influence of instance x_*_.

To describe the quantitative contribution of the observation instance x_*_, the expectation of VAF is defined as the aggregation value of x_*_:

(3) \begin{equation*} v_{a g g}(x_{*})=\int p(x) v^{\prime }(x, x_{*}) d x, \end{equation*}

with *p*(*x*) the probability density function of the variable *x* in the potential samples pool. When the potential training data are given by a limited set *N*, the discrete version of aggregation value is given as

(4) \begin{equation*} \hat{v}_{a g g}(x_{*})=\frac{1}{n} \sum _{x \in N} v^{\prime }(x, x_{*}). \end{equation*}

#### Represent the value of a sample set considering the redundant information

Since the VAF of each instance is defined on the entire feature space, the VAFs of different samples may overlap, which can represent the redundant information explicitly. If two samples are very close to each other, the majority of their VAFs will overlap. Therefore, the VAF of a set can be represented as the ‘union’ of the individual VAFs, just like the union in Boolean geometry operations. Consequently, the VAF of the set *S* = {*x*_1_, *x*_2_, …, *x*_*m*_} is then defined as

(5) \begin{equation*} v^{\prime }(x, S)=v(x) \max \lbrace k(x, x_{1}), \ldots , k(x, x_{m})\rbrace. \end{equation*}

The function can intuitively represent the neighbouring value distribution and show how much value should be aggregated for a sample *x* when the set *S* is available. The quantitative expression, namely the aggregation value of set *S*, is therefore the same:

(6) \begin{equation*} v_{a g g}(S)=\int p(x) v^{\prime }(x, S) d x. \end{equation*}

Similarly, the estimation under a finite number of samples is

(7) \begin{equation*} \hat{v}_{a g g}(S)=\frac{1}{n} \sum _{x \in N} v^{\prime }(x, S). \end{equation*}

Under this definition, closed high-value instances cannot provide high aggregation value. The optimal sample set selection problem can be defined as a submodular optimisation problem [34]:

(8) \begin{equation*} \max _{S \subseteq N} \hat{v}_{a g g}(S). \end{equation*}

A detailed analysis of the submodular property is discussed in the online supplementary material (S4.4).

#### Greedy optimisation of the aggregation value

The greedy algorithm can provide an approximation to the optimal solution of the aforementioned submodular maximisation problem with the guarantee up to a factor of 1 − 1/*e*[35]. Starting from the empty set *S*_0_, the greedy algorithm queries the new sample to maximise the marginal gain $\Delta (e \mid S_{i-1})=\hat{v}_{a g g}(S_{i-1} \cup \lbrace e\rbrace )-\hat{v}_{a g g}(S_{i-1})$. Then iterative set *S*_*i*_ can be obtained as

(9) \begin{equation*} S_{i}=S_{i-1} \cup \Big \lbrace \begin{array}{c}\scriptstyle{\arg \max }\\ \scriptstyle{e}\end{array} \Delta (e \mid S_{i-1})\Big \rbrace . \end{equation*}

Suppose that ℓ points are sampled from the set *N*, and let $S^{*} \in \arg \max _S\lbrace \hat{v}_{a g g}(S):|S| \le k\rbrace$ be an optimal set of size *k*. The approximation bound of this greedy submodular maximisation can be given as [34]

(10) \begin{equation*} \hat{v}_{a g g}(S_{\ell }) \ge (1-e^{-\ell / k}) \hat{v}_{a g g}(S^{*}). \end{equation*}

## DISCUSSION

This research proposed an aggregation-value-based sampling strategy for optimal sample set selection for data-driven manufacturing applications. The proposed method has the appealing potential to reduce labelling efforts for machine learning problems. A novel aggregation value is defined to explicitly represent the invisible redundant information as the overlaps of neighbouring values. The sampling problem is then recast as a submodular maximisation on the aggregation value, which can be solved using the standard greedy algorithm.

Comprehensive experiments on several manufacturing datasets demonstrate the superior performance of the proposed method and appealing potential to reduce labelling efforts. The detailed analysis on the feature distribution and aggregation value interpret the superiority of aggregation-value-based sampling. Four schemes of the value function show the generalisability of the proposed sampling methods. The basic idea of the proposed sampling method is to maximise the aggregation value, while a limitation here is that the greedy optimisation cannot find the globally optimal solution. Therefore, in the future, we will focus on more effective optimising strategies of aggregation value maximisation. Besides, we will also investigate the possibility of aggregation-value-based data generation in transfer learning, physics-informed machine learning and other data-scarcity scenarios.

## DATA AVAILABILITY

The source code of the research is available at https://github.com/code-cl/AV-Sampling.

## Supplementary Material

nwac201_Supplemental_File
