# Supplementary material for: Sampling via the aggregation value for data-driven manufacturing
Source: Natl Sci Rev. 2022 Sep 24;9(11):nwac201. doi: 10.1093/nsr/nwac201 (PMC9646999; doi:10.1093/nsr/nwac201)
Supplement: nwac201_Supplemental_File [file nwac201_supplemental_file.docx]

Supplementary Materials for
**Sampling via aggregation value for**

**data-driven manufacturing**

CONTENTS

[S1 Methods for Shapley value 1](#_Toc109030556)

[S1.1 Approximate calculation method: TMC-Shapley 1](#_Toc109030557)

[S1.2 Modified TMC-Shapley method 2](#_Toc109030558)

[S2 Introduction of tasks 4](#_Toc109030559)

[S2.1 Rolling bearing fault classification 4](#_Toc109030560)

[S2.2 Cifar10 images classification 5](#_Toc109030561)

[S2.3 Tool wear prediction 6](#_Toc109030562)

[S2.4 Thermochemical analysis of composite 7](#_Toc109030563)

[S2.5 Surface measurement and reconstruction 9](#_Toc109030564)

[S3 Supplementary sampling results 11](#_Toc109030565)

[S3.1 Settings of different sampling methods 11](#_Toc109030566)

[S3.2 Sampling results for CWRU HP0 and Tool B2C4 in Scheme A 11](#_Toc109030567)

[S3.3 Detailed settings and robustness analysis for Scheme B 12](#_Toc109030568)

[S4 Supplementary analysis 15](#_Toc109030569)

[S4.1 Characteristic analysis 15](#_Toc109030570)

[S4.2 Sensitivity analysis 18](#_Toc109030571)

[S4.3 Comparison of different kernel functions 19](#_Toc109030572)

[S4.4 Discussion about the sub-modularity 20](#_Toc109030573)

[Reference 21](#_Toc109030574)

# S1 Methods for Shapley value

In this paper, the modified TMC-Shapley method is adopted to calculate the Shapley value of each training data point. We will first introduce the original TMC-Shapley and then the modified TMC-Shapley.

## S1.1 Approximate calculation method: TMC-Shapley

Theoretically, computing the Shapley value requires all the marginal gains, which are exponentially large in the number of training data. That is an unacceptable computational burden and not practical for real application. Consequently, Ghorbani et al. proposed an approximate method to estimate the Shapley value, named truncated Monte Carlo Shapley (TMC-Shapley) [1].

| **Algorithm 1**: TMC-Shapley method |
| --- |
| **Input**: Training dataset $N$ with size $n$, learning algorithm $\mathcal{A}$, and indicator function $\varphi$ |
| **1.** Initialise Shapley value of sample $v_{0}\left( x_{i} \right)=0,$ $i=1,2,\ldots,n$, and $t=0$.  **2.** **while** Convergence criterion not met **do**  2.1. $t\leftarrow t+1$.  2.2. Generate random permutation of training dataset $N$, denoted as $\pi_{t}$.  2.3. $\varphi_{0}^{t}\leftarrow\varphi\left( \emptyset,\mathcal{A} \right)$.  2.4. **for** $j\in\left\{ 1,2,\ldots,n \right\}$ **do**  2.4.1. **if** $\left\vert\varphi\left( N,\mathcal{A} \right)-\varphi_{j-1}^{t} \right\vert\boldsymbol{<}$ Performance Tolerance  $\varphi_{j}^{t}\leftarrow\varphi_{j-1}^{t}$.  2.4.2. **else**  $\varphi_{j}^{t}\leftarrow\varphi\left( \left\{ \pi_{t}\left[ 1 \right],\ldots, \pi_{t}\left[ j \right] \right\},\mathcal{A} \right)$.  **end if**  2.4.3. $v_{t}\left( \pi_{t}\left[ j \right] \right)\leftarrow\frac{t-1}{t}v_{t-1}\left( \pi_{t}\left[ j \right] \right)+\frac{1}{t}\left( \varphi_{j}^{t}-\varphi_{j-1}^{t} \right)$.  **end for**  **end while**  **3.** $v_{t}\left( x_{i} \right)\leftarrow v_{t}\left( \pi_{t}\left[ \mathbf{Index(}x_{i}\mathbf{)} \right] \right), i=1,2,\ldots,n$. |
| **Output**: Shapley value of all training data $v_{t}\left( x_{i} \right),$ $i=1,2,\ldots,n.$ |

The overall process of the TMC-Shapley method is described in Algorithm 1. For the TMC-Shapley method, the Shapley value can be formulated as the following expectation calculation problem, shown in Eq. (1.1).

$$\begin{aligned} v\left( x_{*} \right)=\mathbb{E}_{\pi\sim\Pi}\left[ \varphi\left( S_{\pi}^{*}\cup\left\{ x_{*} \right\} \right)-\varphi\left( S_{\pi}^{*} \right) \right]\#\left( 1.1 \right) \end{aligned}$$

where $\Pi$ is the uniform distribution over all $n!$ possible permutations of training dataset $N$, and $S_{\pi}^{*}$ is the subset of $N$ consisting of all the data points before $x_{*}$ in permutation $\pi$. To estimate the Shapley value, a random permutation of $N$ is firstly generated. Then, the marginal gain $\varphi\left( S_{\pi}^{*}\cup\left\{ x_{*} \right\} \right)-\varphi\left( S_{\pi}^{*} \right)$ for each sample $x_{*}$ can be calculated under the given learning algorithm and indicator function. Repeating the above steps, more marginal gains can be obtained, and the Shapley value of each training data can be simply approximated by the average of all marginal gains. Moreover, the convergence criterion is designed to decide when to stop repeating. Since the marginal gain always approaches zero as the number of training data increases, a performance tolerance parameter is defined to truncate the calculation in each permutation. Then the marginal gain of the following data points in this permutation will be set to zero.

## S1.2 Modified TMC-Shapley method

The experiments show that the performance of the trained model is unstable when the size of the training set is very small. Therefore, we set an initial data size $n_{0}$ before calculating marginal gain, instead of starting with one data point. Moreover, the $\alpha\varphi\left( N, \mathcal{A} \right) \left( 0<\alpha<1 \right)$ is designed as “Performance Tolerance”. Furthermore, the convergence criterion is defined as the maximum variation of all data Shapley value:

$$\begin{aligned} \max_{} \left\{ \frac{1}{k}\sum_{t=m-k}^{m} \left( v_{t}\left( x_{i} \right)-v_{min}\left( x_{i} \right) \right), i=1,2,\ldots,n \right\}<\beta\#\left( 1.2 \right) \end{aligned}$$

| **Algorithm 2**: Modified TMC-Shapley method |
| --- |
| **Input**: Training data $N$ with size $n$, learning algorithm $\mathcal{A}$, and indicator function $\varphi$, $S=20$, $n_{0}$, $\alpha$, $\beta$, $k$. |
| **1.** Initialise data Shapley value $v_{0}\left( x_{i} \right)=0,$ $i=1,2,\ldots,n$, and $t=0$.  **2. for** $s\in\left\{ 1,2,\ldots,S \right\}$ **do**  2.1. $t\leftarrow t+1$.  2.2. Generate a random permutation of training dataset $N$, denoted as $\pi_{t}$.  2.3. Initialize $\varphi_{0}^{t}\leftarrow\varphi\left( \left\{ \pi_{t}\left[ 1 \right],\ldots, \pi_{t}\left[ n_{0} \right] \right\},\mathcal{A} \right)$ and $T\_c=0$.  2.4. **for** $j\in\left\{ n_{0},n_{0}+1,\ldots,n \right\}$ **do**  2.4.1. **if** $\left\vert\varphi\left( N,\mathcal{A} \right)-\varphi_{j-1}^{t} \right\vert\boldsymbol{<}\alpha\varphi\left( N, \mathcal{A} \right)$ and $T\_c>3$:  $\varphi_{j}^{t}\leftarrow\varphi_{j-1}^{t}$, $T\_c\leftarrow T\_c+1$.  2.4.2. **else**  $\varphi_{j}^{t}\leftarrow\varphi\left( \left\{ \pi_{t}\left[ 1 \right],\ldots, \pi_{t}\left[ j \right] \right\},\mathcal{A} \right)$, $T\_c=0$.  **end if**  2.4.3. $v_{t}\left( \pi_{t}\left[ j \right] \right)\leftarrow\frac{t-1}{t}v_{t-1}\left( \pi_{t}\left[ j \right] \right)+\frac{1}{t}\left( \varphi_{j}^{t}-\varphi_{j-1}^{t} \right)$.  **end for**  **end for**  **3.** **if** $\max_{} \left\{ \frac{1}{k}\sum_{t=m-k}^{m} \left( v_{t}\left( x_{i} \right)-v_{min}\left( x_{i} \right) \right), i=1,2,\ldots,n \right\}>\beta$ **return** step 2.  **else** $v_{t}\left( x_{i} \right)\leftarrow v_{t}\left( \pi_{t}\left[ \mathbf{Index(}x_{i}\mathbf{)} \right] \right), i=1,2,\ldots,n$.  **4.** $v_{t}^{'}\left( x_{i} \right)\leftarrow v_{t}\left( x_{i} \right)-\min\left\{ v_{t}\left( x_{i} \right),i=1,2,\ldots,n \right\}, i=1,2,\ldots,n$. |
| **Output**: Shapley value of all training data $v_{t}^{'}\left( x_{i} \right),$ $i=1,2,\ldots,n.$ |

Where $m$ is the total number of iterations, $0<k<m$ is a constant, and the sum $\sum_{t=m-k}^{m}$represents the Shapley value’s variation of the last $k$ iterations, $v_{t}\left( x_{i} \right)$ is the Shapley value of data point $x_{i}$ after completing the $t_{\mathrm{th}}$ iteration, and $v_{min}\left( x_{i} \right)=\min\left\{ v_{t}\left( x_{i} \right), t=m-k,\ldots,m \right\}$. Furthermore, $\beta$ is the defined convergence tolerance.

To ensure the convergence of the Shapley value and reduce the randomness of the training process, the number of iterations increases by $S=20$ until reaching the convergence criterion. The details of the modified TMC-Shapley method are described in Algorithm 2. Notice that, the Shapley value is converted to a non-negative by subtracting the minimum value.

# S2 Introduction of tasks

## S2.1 Rolling bearing fault classification

Bearing failure is one of the foremost causes of breakdown in rotating machinery. Therefore, it is essential to detect the faults timely and precisely before the serious consequences. Typically, bearing faults occur in three components, the rolling elements, the outer race, and the inner race of the bearing. When the bearing is getting close to the end of its lifetime, some faults including cracks and deformation may cause abnormal vibration, which can be captured by accelerometers. Therefore, vibration signal is one of the most reliable and effective information sources for bearing fault diagnosis. This paper focuses on how to sample the “valuable” labelled data for establishing a fault classification model of the rolling bearings, which can potentially reduce the labelling efforts for further fault diagnosis.

The bearing fault dataset provided by Case Western Reserve University (CWRU) data center [2][3] has become the benchmark for the bearing faults diagnosis problem. To prove the effectiveness of our method, experiments are performed based on the drive end bearing fault data at 12K sampling frequency. This dataset consists of the ordinary situation and nine fault situations under three fault diameters (0.1778mm, 0.3556mm and 0.5334mm) and three fault locations (inner race, rolling element and outer race). The vibration signals of normal and faulty conditions are collected under different motor loads (HP=0,1,2,3). Details of this dataset include the following:

- The following experiments consist of two typical predictions on (HP=0,1), and two cross-task situations HP0$\to$HP1 and HP1$\to$HP0. Cross-task means that the value function learnt from the source task is applied to the target task.
- The frequency resolution of this data set (12 kHz) is sufficiently high to obtain a large quantity of time-course measurements for classification. Assuming the faulty signal is stationary, we use a sliding window with a length of 1024 to reshape the original data and obtain 1539 reconstructed samples. The dimension of feature data (HP0 and HP1) is reduced to 256 by a 1D CNN for simplification. The structure of 1D CNN is shown in Fig. S 2.1.
- The dataset is randomly split into two parts: 50% for training (770 samples), and the rest 50% for the test (769 samples).


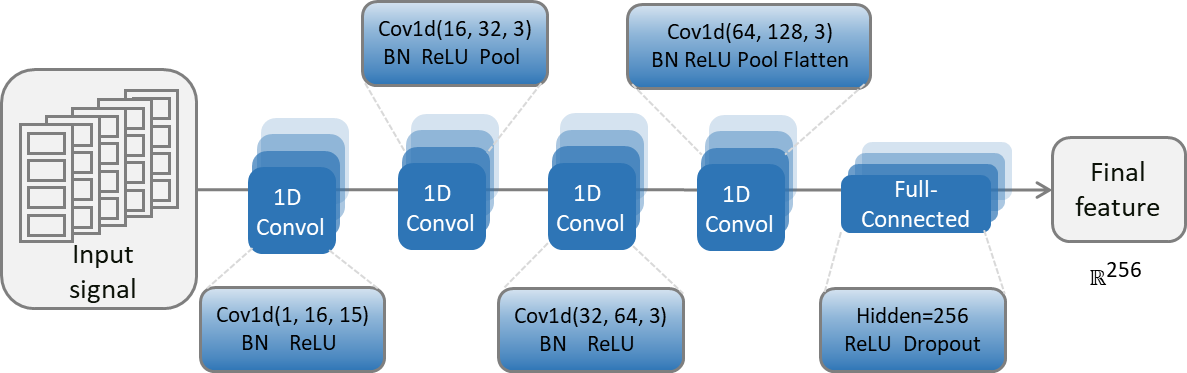


Fig. S 2.1 The structure of 1D CNN.

For the calculation of Shapley value, the learning algorithm $\mathcal{A}$ is logistic regression (LGR), the indicator function $\varphi$ is the accuracy of classification (ACC), and the parameters $n_{0}=10$, $\alpha=0.02$, $\beta=0.002$, and $k=50$. In addition, the kernel width $\sigma$ of the value aggregation function is set to 100.

## S2.2 Cifar10 images classification

The Cifar10 dataset consists of 60000 32x32 colour images in 10 classes, with 6000 images per class [4]. There are 50000 training images and 10000 test images. The dataset is divided into five training batches and one test batch, each with 10000 images. The test batch contains exactly 1000 randomly selected images from each class. The training batches contain the remaining images in random order, but some training batches may contain more images from one class than another. Between them, the training batches contain exactly 5000 images from each class.

Since the proposed sampling method focuses on the data scarcity scenario, the original Cifar10 dataset is too large. We use the test batch as a simplified dataset and split it into training and test data, with 10% and 90% respectively.

We select five categories among ten classes and simplify the 10-way task to a 5-way task. To compute the Shapley value effectively, the size of raw input in the Cifar10 dataset is reduced to a 64-dimensional vector via typical ResNet. The structure of the ResNet is shown in Fig. S 2.2.

For the calculation of Shapley value, the learning algorithm $\mathcal{A}$ is logistic regression (LGR), the indicator function $\varphi$ is the accuracy of classification (ACC), and the parameters $n_{0}=10$, $\alpha=0.01$, $\beta=0.01$, and $k=50$. In addition, the kernel width $\sigma$ of the value aggregation function is set to 10.


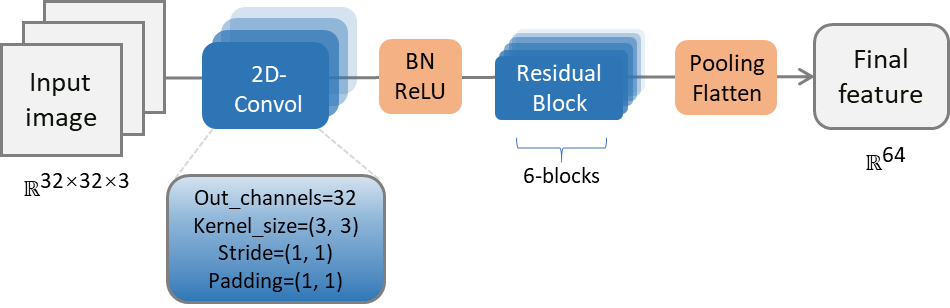


Fig. S 2.2 The structure of ResNet.

## S2.3 Tool wear prediction

The healthy condition of cutting tools can influence the surface texture and precision of the workpiece through direct contact [5]. Recent studies show that only 50%~80% of the tool life is reasonably used, and around 10%~40% of the total machine downtime is wasted on the abnormality of cutting tools. Previous researchers used approximate analytical models to predict tool wear by simplifying the complexity of the machining process. Recently, data-driven methods that build the relationship between the monitoring sensor signals and tool wear value have received much attention. However, the performance of the prediction model relies on sufficient labelled tool wear data, which is usually expensive and time-consuming. This paper focuses on how to sample limited “valuable” tool wear data to establish a data-driven model.

The tool wear dataset from the 2010 PHM society conference data challenge [6][7] comprises six individual cutter records. C1, C4 and C6 are training data. C2, C3 and C5 are test data. Each training data record contains seven sensor signals and the wear values of the three blades of the milling cutter. Details of this dataset include the following:

- Each training record contains one “wear” file that lists wear after each cut, and a folder with approximately 300 individual data acquisition files (one for each cut).
- Seven types of sensor signals correspond to: Force (N) in the X dimension, Force (N) in the Y dimension, Force (N) in the Z dimension, Vibration (g) in the X dimension, Vibration (g) in the Y dimension, Vibration (g) in the Z dimension, RE-RMS(V).
- Two regression tasks are formulated, blade No. 2 of cutting tool No. 4 and blade No. 3 of cutting tool No. 6, B2C4 and B3C6 for short. We also select the x-axis milling force signal as input. Since the dimension of the raw signal exceeds 200,000 dimensions and the length is not uniform, we perform uniform down-sampling and reduce the dimensionality to 20,000. Then further dimensionality reduction is carried out by a convolutional neural network, as presented in Fig. S 2.3. We choose the feature map with 128 dimensions produced by the first full-connected layer (FC-1) as the final feature to compute the Shapley value. Note that the feature map comes from *Epoch 0*, e.g. the network has not been iteratively trained.
- The obtained dataset is randomly split into two parts: 70% for training (219 samples), and the rest 30% for the test (94 samples).

For the calculation Shapley value, the learning algorithm $\mathcal{A}$ is Gaussian process regression (GPR), the indicator function $\varphi$ is the mean absolute error (MAE), and the parameters $n_{0}=1$, $\alpha=0.2$, $\beta=1$, and $k=20$. In addition, the kernel width $\sigma$ for In addition, the kernel widths $\sigma$ of the value aggregation function are 100 and 50 for task B2C4 and B3C6 respectively.


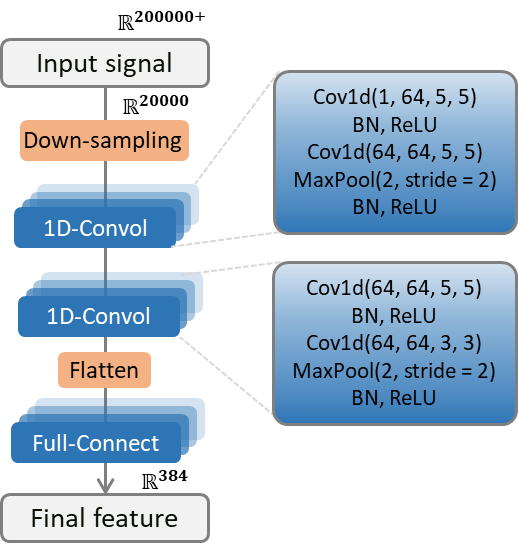


Fig. S 2.3 Raw data dimensionality reduction and CNN structure.

## S2.4 Thermochemical analysis of composite

The curing temperature cycles have a significant influence on the mechanical properties of composite workpieces. The thermal lag value is defined as the maximum difference between air temperature and any point in the part thickness during the heat-up step. It is often used as an optimisation target to guide the design of temperature cycles in actual production. Traditional numerical methods, finite element method (FEM) and finite difference method (FDM), can simulate the thermal lag of the given temperature cycle, but the high computational cost of numerical methods limits their further application. Recently, researchers have tried to build data-driven surrogate models to accelerate process optimization [8]. Considering a 1D exothermic heat transfer situation where an AS4/8552 composite part with a thickness of $L_{c}=20mm$, is placed on an Invar tool with a thickness of $L_{t}=30mm$. The material properties for AS4 fiber, 8552 epoxy and Invar tool are listed in Table 2.1. Given the densities and specific heat capacity of fiber and resin, the density $\rho_{c}$ and specific heat capacity $C_{c}$ of composite can be calculated via rules of the mixtures, i.e. Eq. (2.1) and Eq. (2.2). The thermal conductivity of composite in the thickness direction can be obtained from the Springer-Tsai model, as shown in Eq. (2.3), Eq. (2.4) and Eq. (2.5). Considering the complex flow field inside autoclaves, we assume convective boundary conditions. As presented in Fig. S 2.4, we design a type of one-hold cure cycle, which can be characterised by heating rate, holding time, holding temperature and cooling rate.

Table 2.1 Material properties for AS4 fiber, 8552 epoxy and Invar tool.

|  | Volume fraction, $v$ | Density, $\rho\left（ kg/m^{3} \right）$ | Specific heat capacity, $C (J/kg K)$ | Thermal conductivity, $k (W/m K)$ |
| --- | --- | --- | --- | --- |
| AS4 fiber | $v_{f}=0.574$ | $\rho_{f}=1790$ | $C_{f}=914.0$ | $k_{f}=3.960$ |
| 8552 resin | $v_{r}=0.426$ | $\rho_{r}=1300$ | $C_{r}=1304.2$ | $k_{r}=0.212$ |
| Invar tool | — | $\rho_{t}=8150$ | $C_{t}=510.0$ | $k_{t}=13.0$ |

$$\begin{aligned} \rho_{c}=\rho_{r}v_{r}+\rho_{f}v_{f}\#\left( 2.1 \right) \end{aligned}$$

$$\begin{aligned} C_{c}=C_{r}v_{r}+C_{f}v_{f}\#\left( 2.2 \right) \end{aligned}$$

$$\begin{aligned} k_{c}=k_{r}\left( \left( 1-2\Omega\right)+\frac{1}{\Gamma}\left( \pi-\frac{4}{\Upsilon}{tan}^{-1} \left( \frac{\sqrt{1-\Gamma^{2}\Omega^{2}}}{1+\Omega\Gamma} \right) \right) \right)\#\left( 2.3 \right) \end{aligned}$$

$$\begin{aligned} \Gamma=2\left( \frac{k_{r}}{k_{f}}-1 \right)\#\left( 2.4 \right) \end{aligned}$$

$$\begin{aligned} \Omega=\sqrt{\frac{v_{f}}{\pi}}\#\left( 2.5 \right) \end{aligned}$$

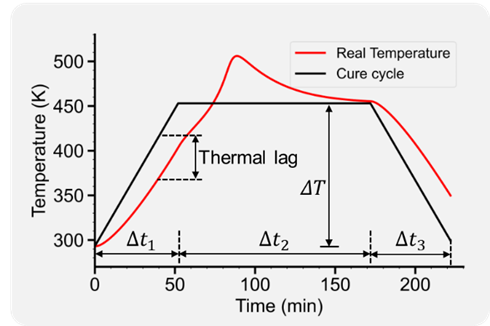


Fig. S 2.4 The one-hold cure cycle and the designing variables.

As mentioned above, the input feature of thermochemical analysis of composite data is composed of 6 variables, including heating rate $H_{rate}$, the cooling rate $C_{rate}$, the hold temperature $t_{h}$, the hold time $t_{h}$, and both-sides heat transfer coefficients $h_{c}$ and $h_{t}$.

Table 2.2 Detailed variable ranges of the temperature cycle.

| Parameters | Range | Inputs | Range |
| --- | --- | --- | --- |
| $\Delta t_{1}$ | $\left( 30,160 \right)/min$ | $h_{c}$ | $\left( 20,100 \right)/(W/m^{2}K)$ |
| $\Delta t_{2}$ | $\left( 30,150 \right)/min$ | $h_{t}$ | $\left( 20,100 \right)/(W/m^{2}K)$ |
| $\Delta t_{3}$ | $\left( 60,150 \right)/min$ | $H_{rate}$ | $\Delta T/\Delta t_{1}$ |
| $\Delta T$ | $\left( 150,175 \right)/K$ | $t_{h}$ | $\Delta t_{2}$ |
| $T_{0}$ | $293$K | $T_{h}$ | $T_{0}+\Delta T$ |
|  | | $C_{rate}$ | $\Delta T/\Delta t_{3}$ |

For the Scheme A, we randomly sample from the parameter ranges given in Table 2.2. to generate 1500 temperature cycles and use FEM to calculate the thermal lag value as the corresponding outputs. The obtained dataset is then randomly split into two parts: 600 samples for training, and 900 samples for the test.

For Scheme B, we generate 600 potential curing parameters from a reasonable range and calculate the corresponding thermal lags using a simplified forward finite difference model based on the partial differential equations of heat transfer equation and ordinary differential equations of the cure of degree [8]. The low-fidelity model is not accurate enough but very effective. The 600 low-fidelity data will be used to calculate the value function and serve for the parameters designing for the time-consuming FEM simulation.

Since the dimension of the input feature is only 6, the basic machine learning model is Gaussian Process Regression (GPR) based on the GPyTorch package. The likelihood we used is *gpytorch. likelihoods.GaussianLikelihood.* The mean module and kernel module are *gpytorch.means.ConstantMean()* and *gpytorch.kernels.ScaleKernel (gpytorch.kernels.RBFKernel())* respectively.

For the calculation of Shapley value, the learning algorithm $\mathcal{A}$ is Gaussian process regression (GPR), the indicator function $\varphi$ is the mean absolute error (MAE), and the parameters $n_{0}=5$, $\alpha=0.3$, $\beta=3$, and $k=20$. In addition, the kernel width $\sigma$ is set to 100 in the value aggregation function of the proposed aggregation-value sampling.

## S2.5 Surface measurement and reconstruction

Freeform surfaces are widely used in automation, aerospace, and other industry fields. Mechanical touch probe can provide accurate measurement results to reconstruct the surface for quality assessment or manufacturing process optimisation. However, it is usually time-consuming to collect sufficient probe points for surface reconstruction. An efficient sampling method should enable the reconstruction of the surface under the required accuracy with a limited amount of measurement points. The MATLAB peaks surface is adopted as the simulation case. The function is defined as:

$$\begin{aligned} \begin{aligned} z=6\left( 1-\frac{x}{16} \right)^{2}\cdot\exp\left( -\left( \frac{x}{16} \right)^{2}-\left( \frac{y}{16}+1 \right)^{2} \right)- \\ 20\left( \frac{x}{5\times16}-\left( \frac{x}{16} \right)^{3}-\left( \frac{y}{16} \right)^{5} \right)\cdot\exp\left( -\left( \frac{x}{16} \right)^{2}-\left( \frac{y}{16} \right)^{2} \right) \\ -\frac{2}{3}\exp\left( -\left( \frac{x}{16}+1 \right)^{2}-\left( \frac{y}{16} \right)^{2} \right),x,y\in[-40,40] \end{aligned}\#\left( 2.6 \right) \end{aligned}$$

The function is defined as the nominal surface without noise. The value function is simply defined as the curvature of the surface, which is evaluated using the python library *pyntcloud*. Then, the set of the potential data pool consists of 900 points uniformly sampled from the Peaks function integrating a Gaussian noise $\sigma=0.02\mu m$ as the simulated probe data. Another set with 1600 points integrating a Gaussian noise $\sigma=0.02\mu m$ is defined as the test set to evaluate the performance of the reconstructed surface.

The basis learner for surface reconstruction is also Gaussian Process Regression (GPR) based on the GPyTorch package. The likelihood we used is *gpytorch. likelihoods.GaussianLikelihood.* The mean module and kernel module are *gpytorch.means.ConstantMean()* and *gpytorch.kernels.ScaleKernel (gpytorch.kernels.RBFKernel())* respectively.

# S3 Supplementary sampling results

## S3.1 Settings of different sampling methods

The proposed sampling method HighAV and LowAV are compared with the random sampling method (Random), the cluster sampling in data feature space (Cluster), and the Shapley value-based sampling (HighSV).

**Random**: Random sampling is a widespread technique that randomly selects $m$ samples from a data pool $N$. The function *random.sample* of Python package is adopted here.

**Cluster**: The clustering for data feature is the most intuitive representativeness-based sampling method. In this section, the K-means clustering algorithm is used to find $m$ cluster centers of feature space, then the nearest data point of each cluster center is sampled by the Nearest Neighbors method to form a dataset of size $m$. The *KMeans* and *NearestNeighbors* of Python package *sklearn* are used to carry out the whole process, where “init = k-means++” and “n_init = 10” are set for the function *KMeans*, and “n_neighbors = 1” and “algorithm = ball_tree” are set for the function *NearestNeighbors*.

**HighSV**: Sampling $m$ data points with the highest Shapley value. As discussed in section 1, given a fixed dataset, the learning algorithm $\mathcal{A}$, indicator function$\varphi$, and the parameters $n_{0}$, $\alpha$, $\beta$, $k$ need to be determined before calculating data Shapley value.

Computing data Shapley value requires the prediction performances of different subsets, so the accuracy of the Shapley value depends heavily on the stability of the learning model $\mathcal{A}$. To reduce the randomness, the logistic regression (LGR) and Gaussian process regression (GPR) are selected as the learning model $\mathcal{A}$ for classification and regression problem respectively.

For the regression tasks, we use Gaussian Process Regression (GPR) to establish prediction models based on the GPyTorch package. The likelihood we used is *gpytorch. likelihoods.GaussianLikelihood.* The mean module and kernel module are *gpytorch.means.ConstantMean()* and *gpytorch.kernels.ScaleKernel (gpytorch.kernels.RBFKernel())* respectively. We set the multivariate normal distribution to *gpytorch.distributions.MutivariteNormal.* As for the training process, we use Adam optimiser with a learning rate of 0.1 and an iteration of 100.

For the classification task, we use the Logistic Regression (LR) algorithm to establish prediction models. LR algorithm is simple, efficient and stable, which is very suitable for verifying sampling methods. Here we use *LogisticRegression* from *sklearn.linear_model*.

## S3.2 Sampling results for CWRU HP0 and Tool B2C4 in Scheme A

The sampling results of CWRU HP1, Composite, Cifar10 and Tool wear B3C6 were reports in the manuscript Scheme A. The sampling results of the rest two case, CWRU HP0 and Tool wear B2C4, are shown as Fig. S 3.1.

| 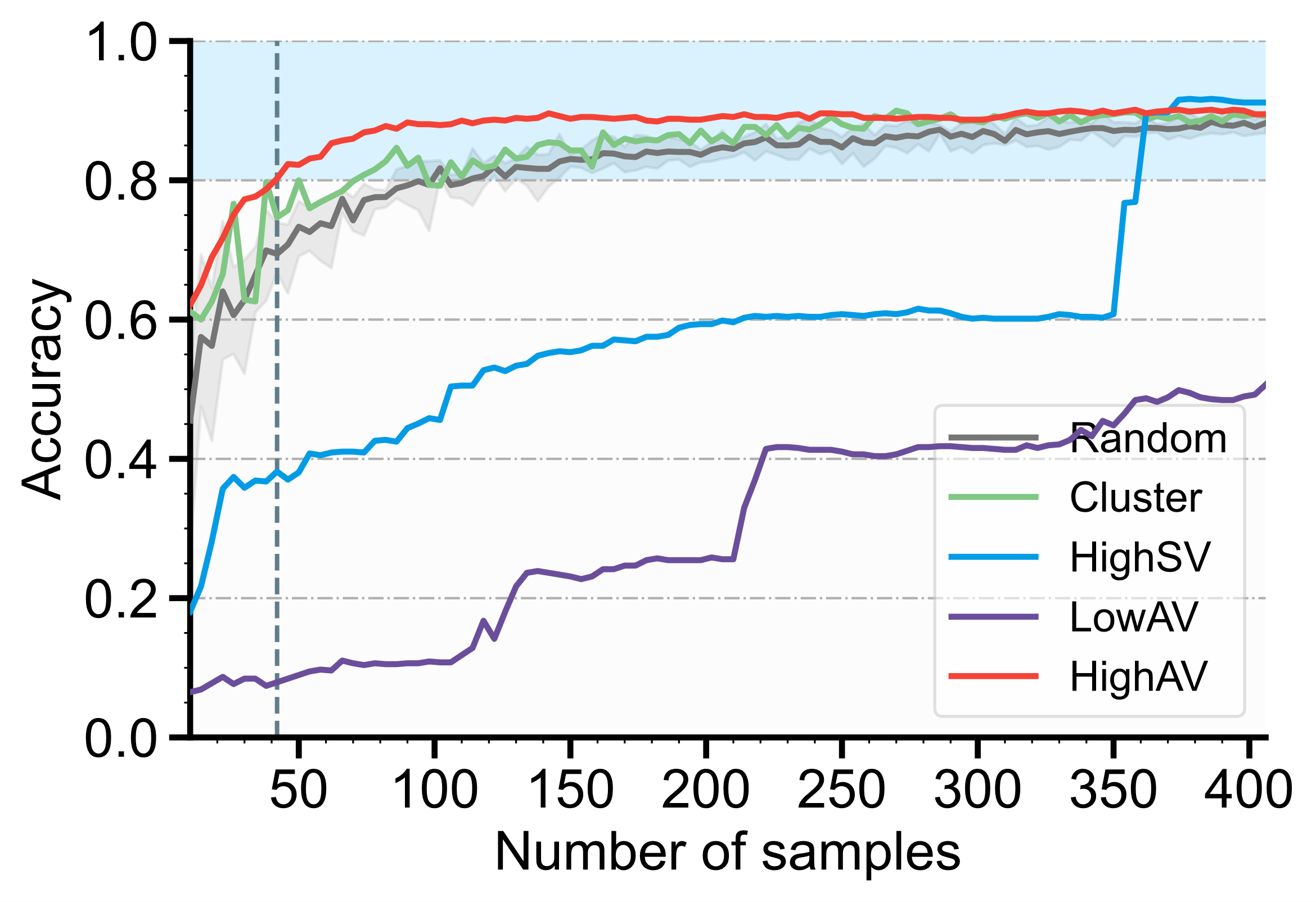 | 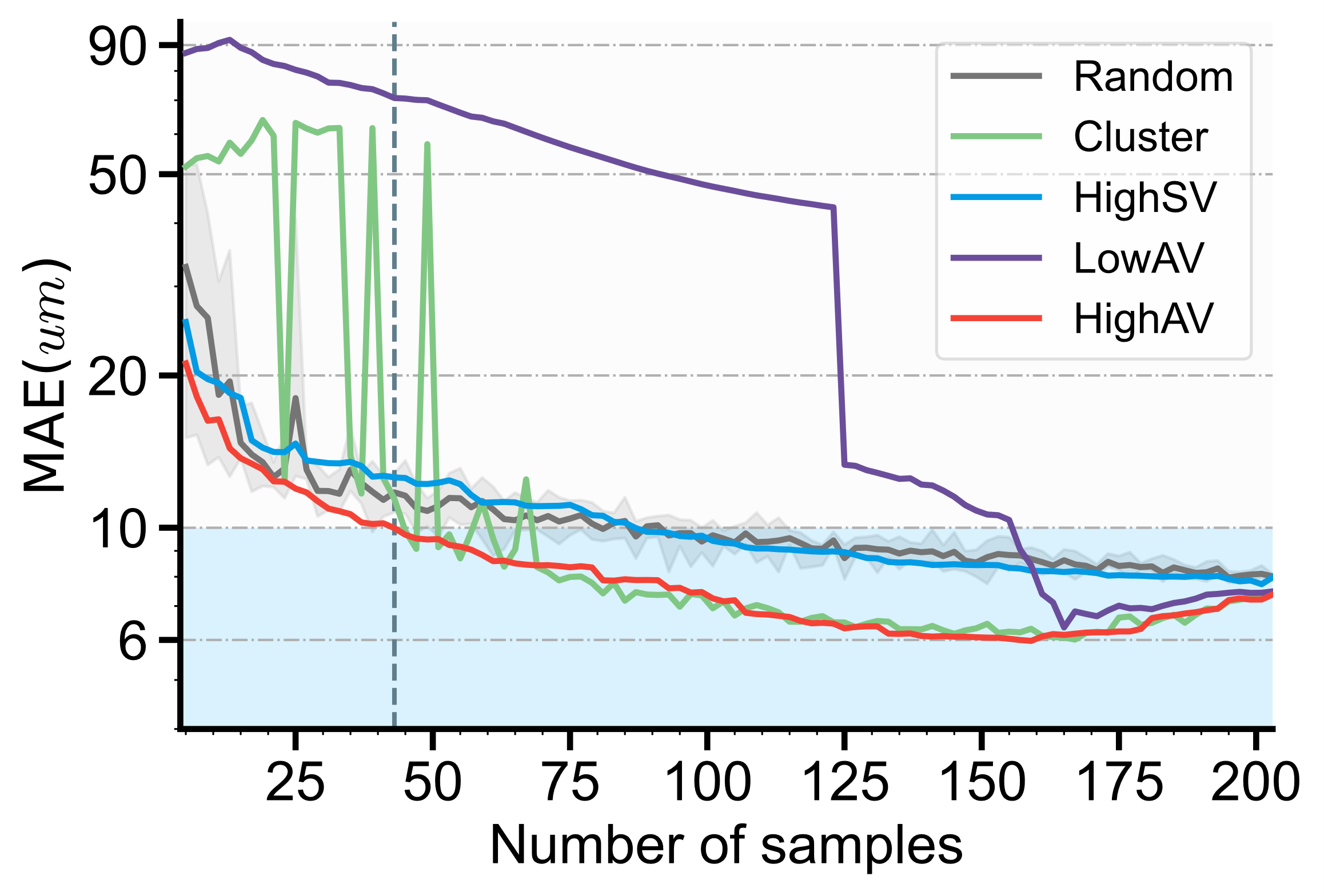 |
| --- | --- |
| (a) | (b) |

Fig. S 3.1 (a) Sampling results on CWRU HP0. (b) Sampling results on Tool wear B2C4.

## S3.3 Detailed settings and robustness analysis for Scheme B

In this section, the cross-tasks HP0$\to$HP1, HP1$\to$HP0 on CWRU and B2C4$\to$B3C6, B3C6$\to$B2C4 on Wear are considered to investigate the possibility of reusing the value function learnt from the similar task to the target task.

To guide the cross-task sampling, we introduce a multiple-layer perceptron model called *MLPRegressor* from *sklearn.neural_network* to model the value function. We set 3 hidden layers with different width, which contains two settings: [20, 30, 20] and [20, 40, 20]. The “ReLU” is chosen as the activation function. The solver is “*lbfgs*”, an optimiser in the family of quasi-Newton methods. Other parameters are set by default.

### S3.3.1 Classification task: CWRU HP0 and HP1

There are two cross-tasks HP0$\to$HP1 and HP1$\to$HP0 on the CWRU dataset, the corresponding value functions are learned from the trained Shapley value in Scheme A by NN with the neuron numbers of hidden layers 20, 30, and 20. Moreover, the kernel widths are set to 100 for two cross-tasks in the aggregation-value sampling.

The classification accuracies with different numbers of samples from all methods on cross-tasks HP0$\to$HP1 and HP1$\to$HP0 are shown in Fig. S 3.2a-b respectively. The numbers of samples are selected from 10 to 406 at intervals of 4 one by one.

| 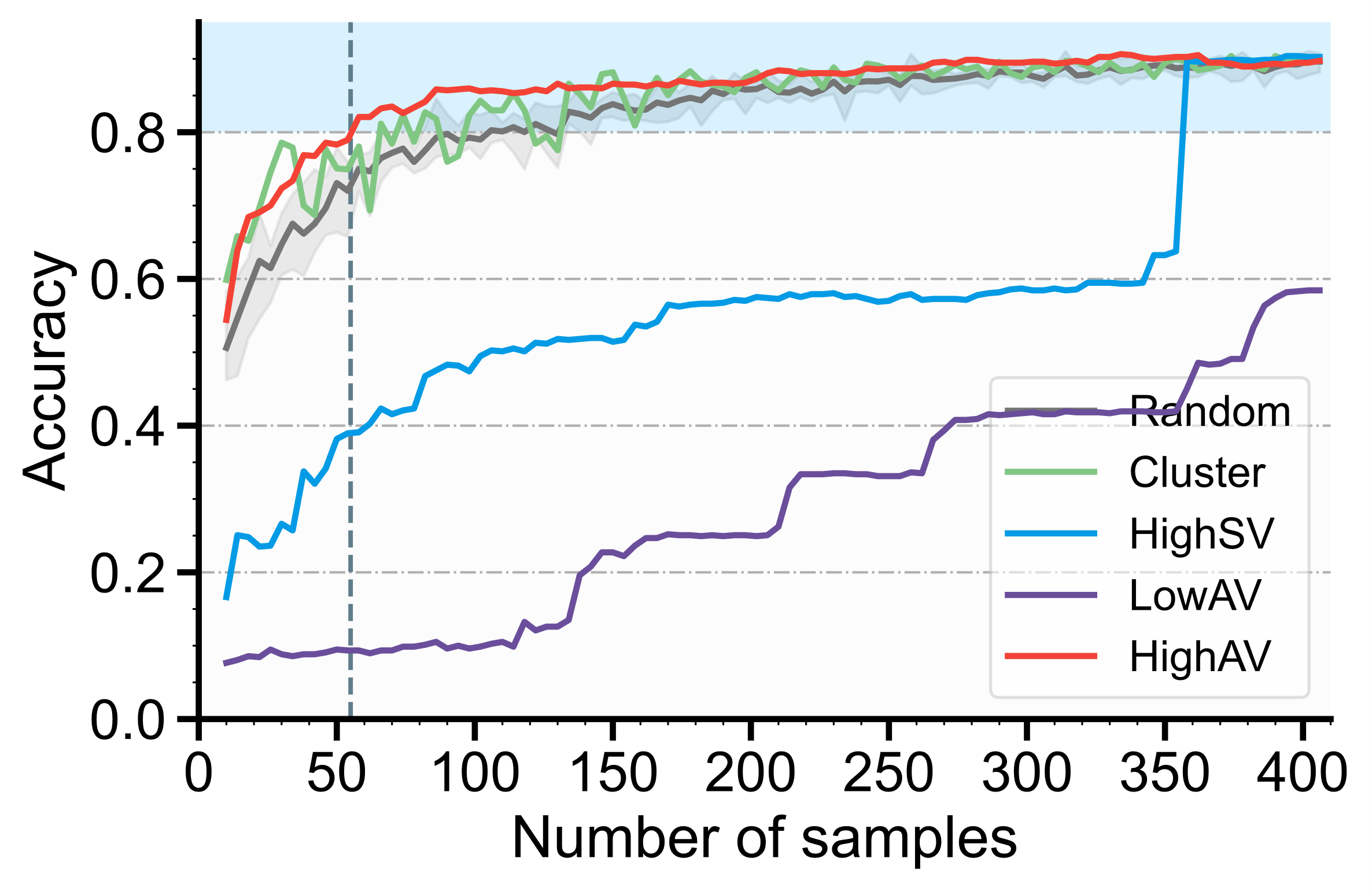 | 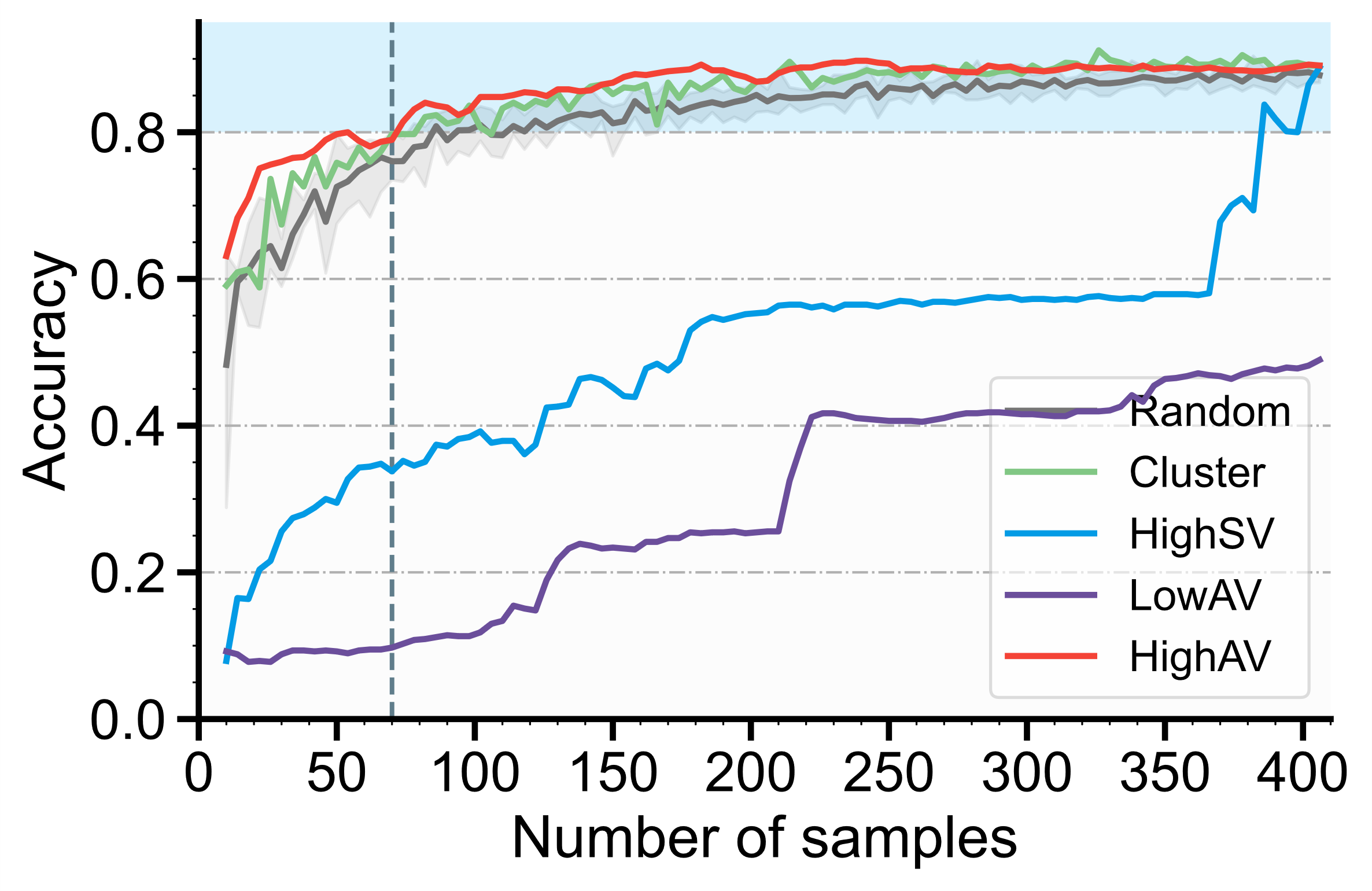 |
| --- | --- |
| (a) | (b) |

Fig. S 3.2 (a) Results of reusing value function of task CWRU HP0 to HP1. (b) Results of reusing value function of task CWRU HP1 to HP0.

The 5 repeating Shapley value results of HP0 and HP1 are used to guide the target tasks HP1 and HP0 respectively, in which the kernel widths $\sigma$ for aggregation-value sampling are set to 100. The means and uncertainty boundaries of 5 MAEs for different methods on tasks HP0$\to$HP1 and HP1$\to$HP0 are shown in Fig. S 3.3a and Fig. S 3.3b respectively. The results indicate that HighAV and LowAV are more stable than HighSV, and HighAV can always provide excellent performances for both cross-tasks HP0$\to$HP1 and HP1$\to$HP0.

| 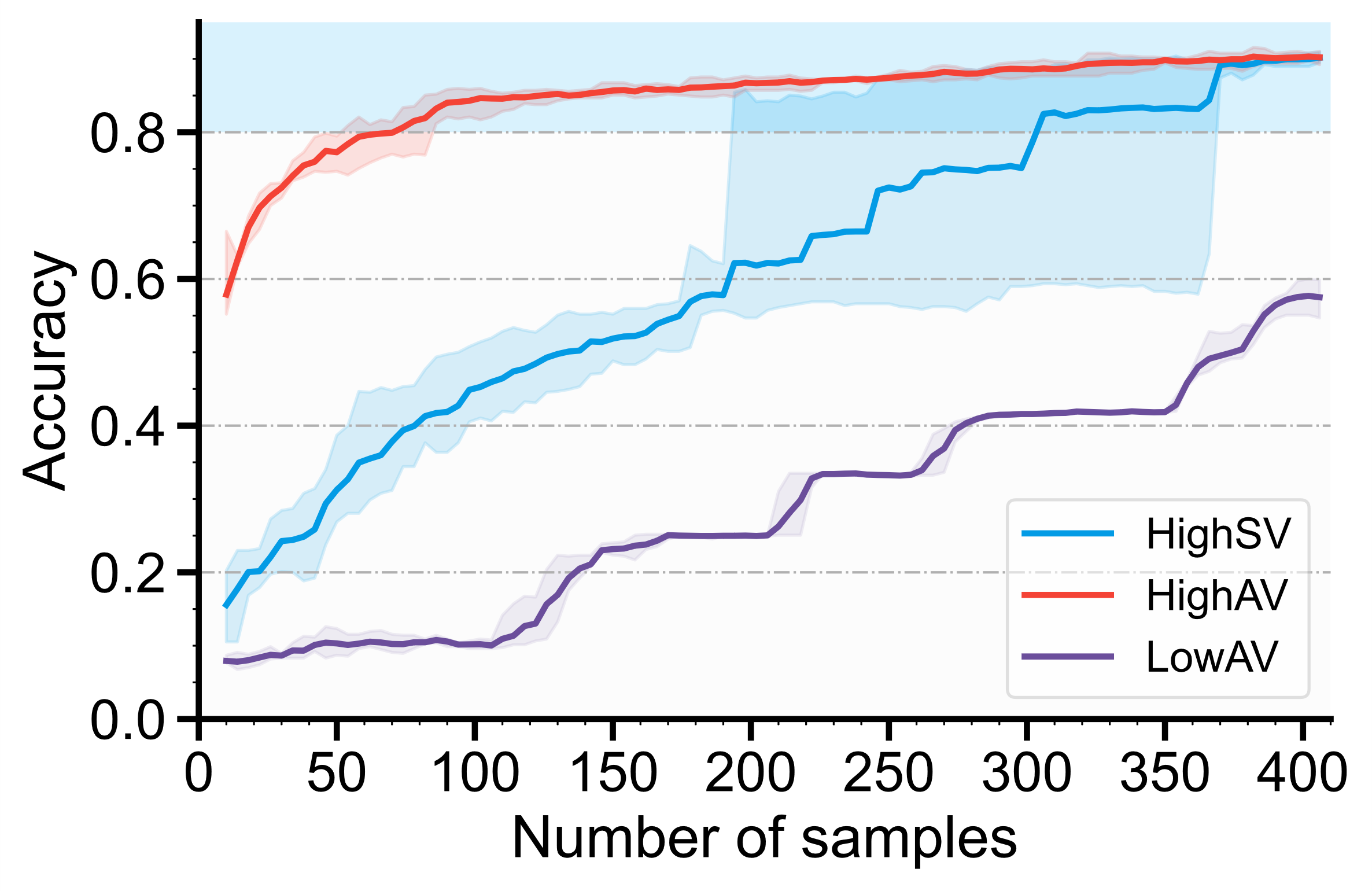 | 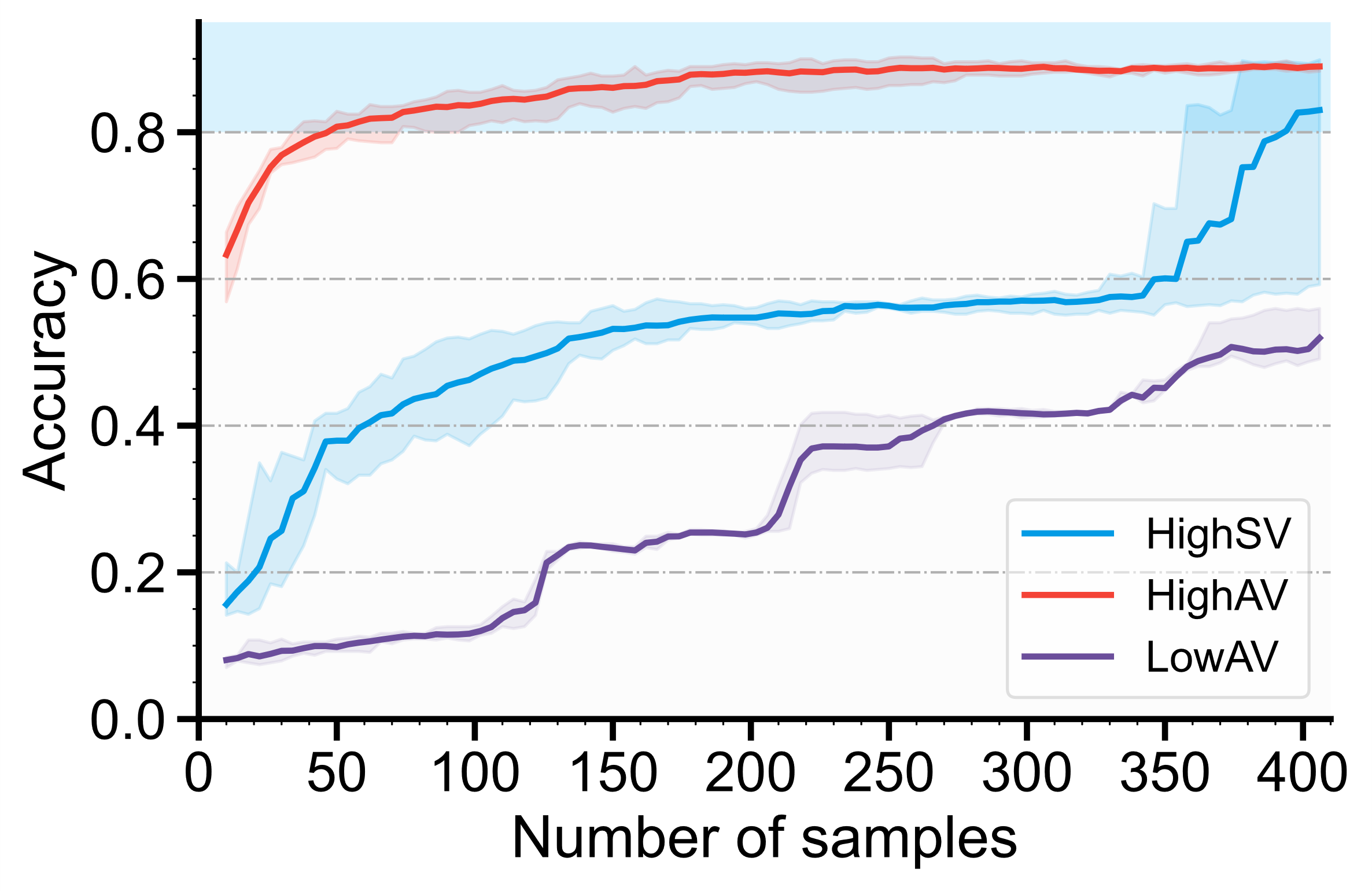 |
| --- | --- |
| (a) | (b) |

Fig. S 3.3 The sensitivity analysis. (a) Sensitivity on the random error of Shapley value on the CWRU HP0$\boldsymbol{\to}$HP1 task. (b) Sensitivity on the random error of Shapley value on the CWRU HP1$\boldsymbol{\to}$HP0 task.

### S3.3.2 Regression task: Wear B2C4 and B3C6

There are two cross-tasks B2C4$\to$B3C6 and B3C6$\to$B2C4 on the Wear dataset, the corresponding value functions are learned from the trained Shapley value in Scheme A by NN with the neuron numbers of hidden layers 20, 40, and 20. Due to the large difference of Shapley value between different data points, there is a challenge for directly modelling the Shapley value function. Therefore, the data feature is first normalised by the function *StandardScaler* in *sklearn*. The log function is further used to reduce the Shapley value difference to ensure the relative value. Finally, the data points with too small Shapley value are deleted. The kernel widths are set to 100 for two cross-tasks in the aggregation-value sampling.

The MAEs with different numbers of samples from all methods on Task B2C4$\to$B3C6 and B3C6$\to$B2C4 are shown in Fig. S 3.4a-b, respectively. The numbers of samples are selected from 5 to 203 at intervals of 2 one by one.

| 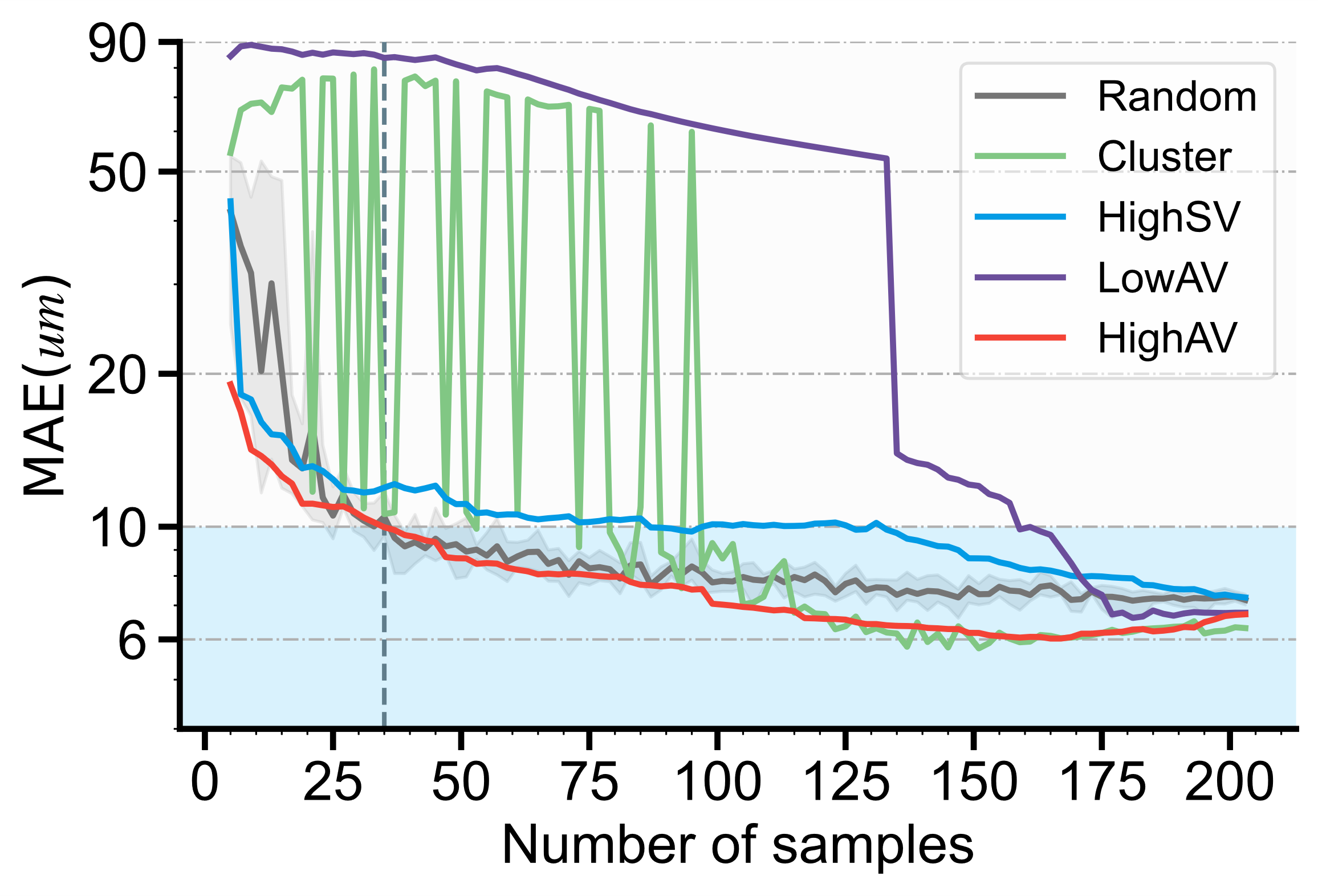 | 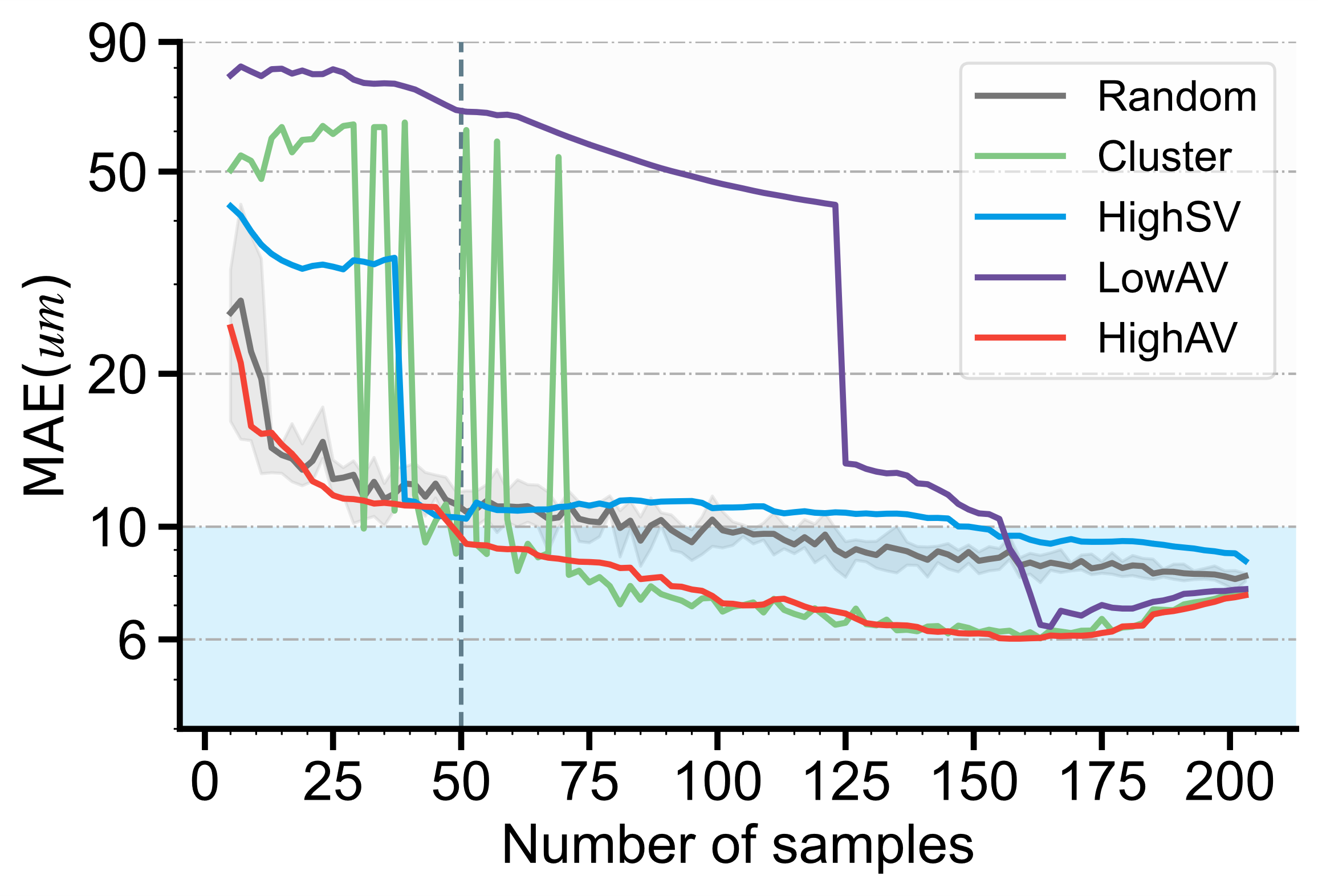 |
| --- | --- |
| (a) | (b) |

Fig. S 3.4 (a) Results of reusing value function of task B2C4 to B3C6. (b) Results of reusing value function of task B3C6 to B2C4.

The 5 repeating Shapley value results of B2C4 and B3C6 are used on target tasks B3C6 and B2C4 respectively, in which the kernel widths $\sigma$ for calculating aggregation value are set to 100 for cross-tasks B2C4$\to$B3C6 and B3C6$\to$B2C4. The means and uncertainty boundaries of 5 MAEs for different methods on tasks B2C4$\to$B3C6 and B3C6$\to$B2C4 are shown in Fig. S 3.5a and Fig. S 3.5b respectively. The results indicate that HighAV is more stable performance than HighSV in most cases, but HighAV shows significant fluctuation on cross-task B2C4$\to$B3C6 with number of samples less than 25.

| 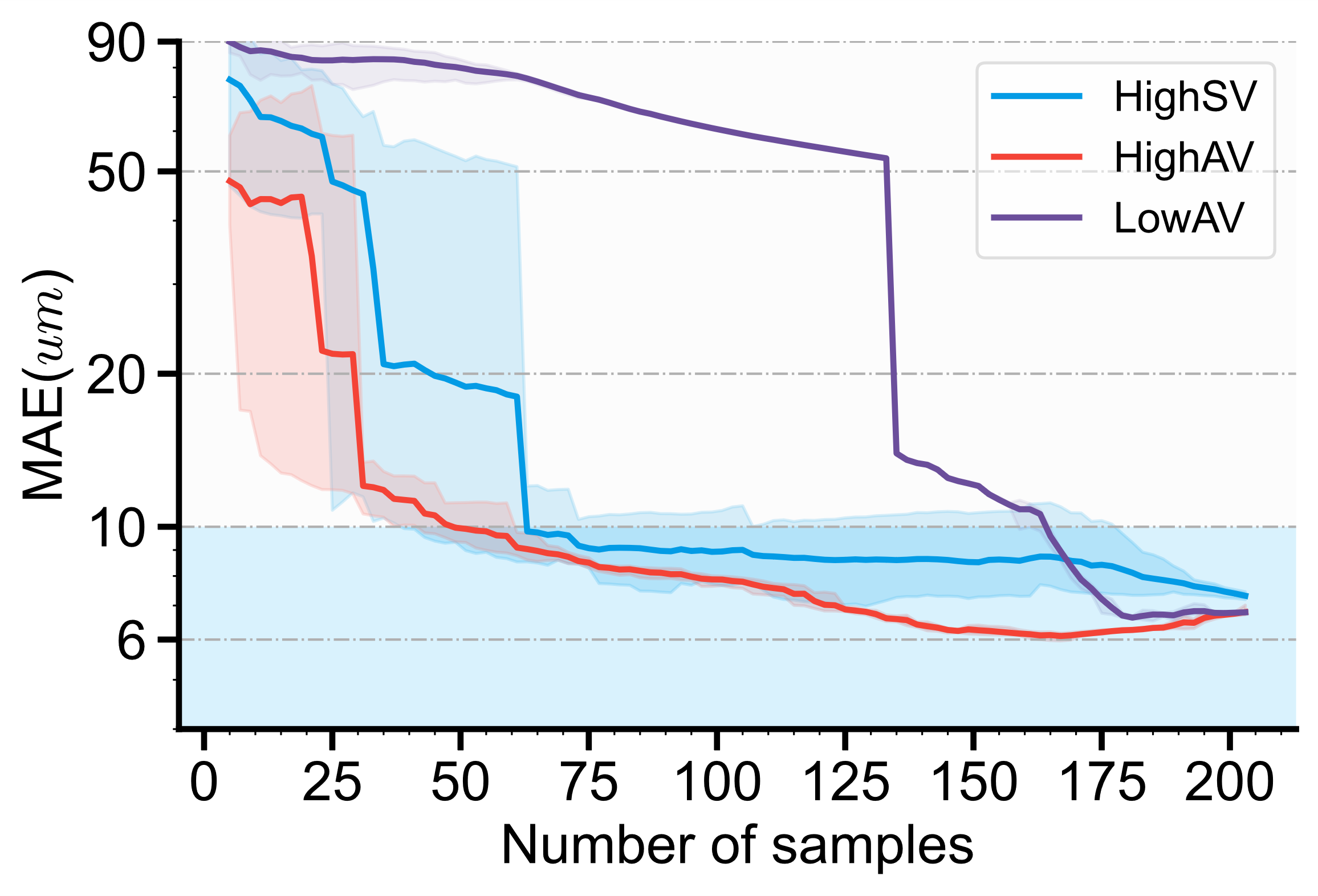 | 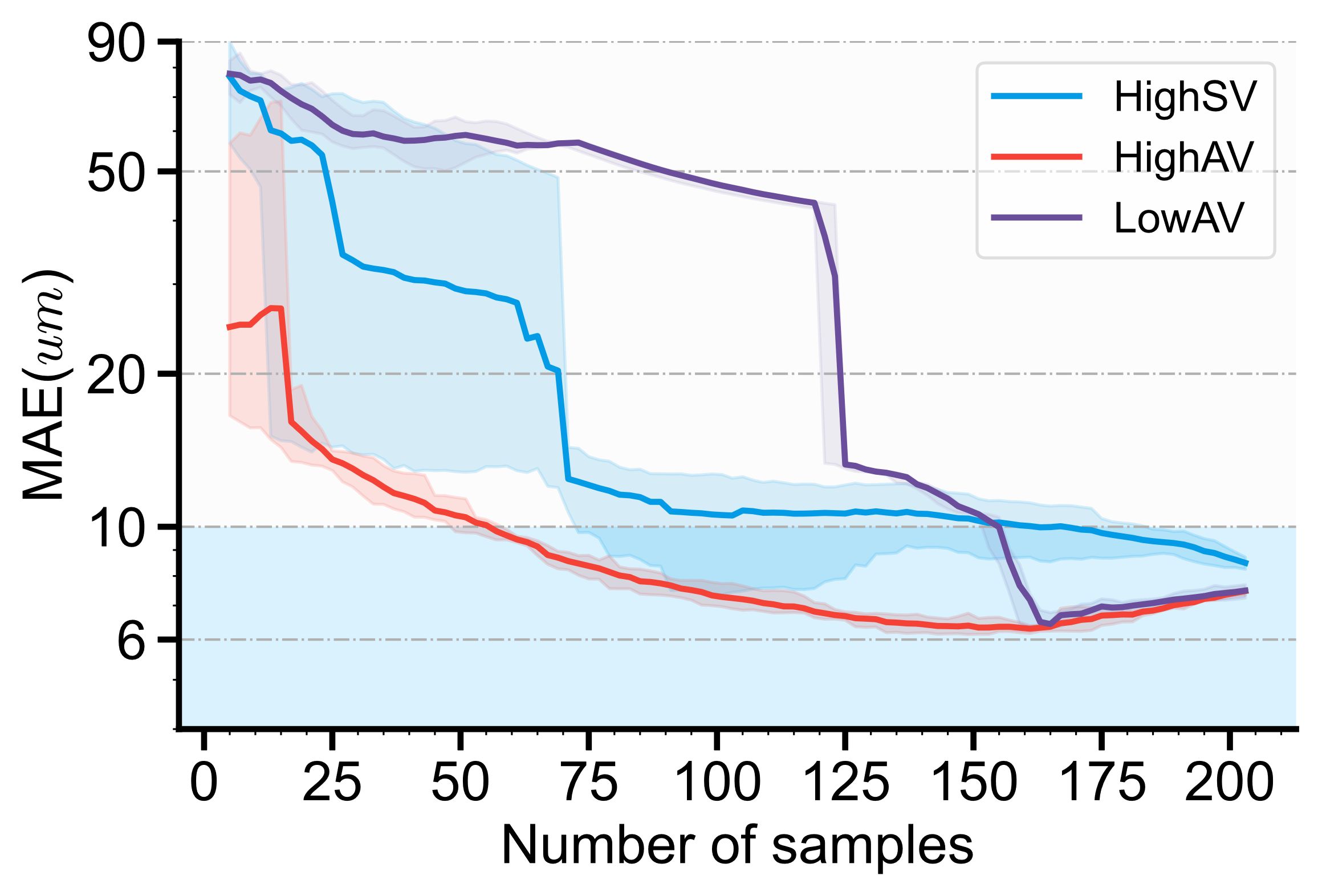 |
| --- | --- |
| (a) | (b) |

Fig. S 3.5 The sensitivity analysis. (a) Sensitivity on the random error of Shapley value on the Wear B2C4$\boldsymbol{\to}$B3C6 task. (b) Sensitivity on the random error of Shapley value on the Wear B3C6$\boldsymbol{\to}$B2C4 task.

# S4 Supplementary analysis

## S4.1 Characteristic analysis

Since the characteristic analysis of the composite task was given in the manuscript. This section will report the characteristic analysis for the rest 5 tasks. Each figure consists of five sub-figures (a-e). Sub-figures a-c report the visualised t-SNE feature distributions of 3 sample sets, generated by HighSV, HighAV and Cluster respectively. 300 green points are randomly selected from the potential data pool, and blue points are samples in the generated sample sets. The red backgrounds in Sub-figures a-c represent the field of Shapley value, generated by modelling the relationship between the visualised feature and the corresponding Shapley value. The darker colour means a larger Shapley value. The label distributions under different numbers of samples are shown in Sub-figures d, the abscissa means the multiple categories for classification problems or multiple intervals of the labels for regression problems, and the red cross represents the vacancy of label in that interval. These results show that HighAV can cover all the label intervals with only a few samples. The comparison between aggregation value and the sum of Shapley value are shown in Sub-figures e.


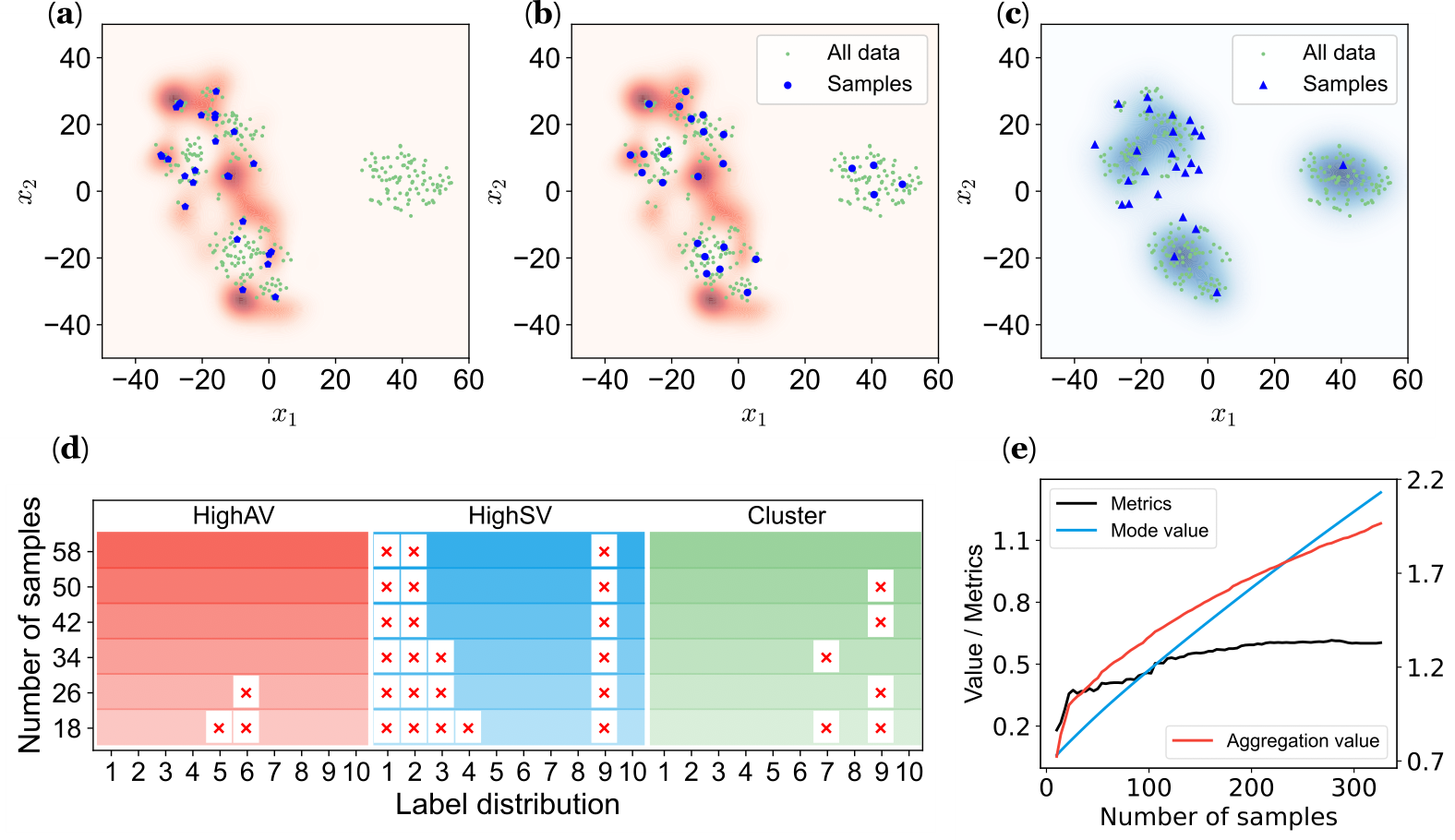


Fig. S 4.1 Characteristics analysis of the CWRU HP0 task. (a) A sample set generated by HighSV. The red background represents the field of Shapley value and the darker colour means a larger value. (b) A sample set generated by HighAV. (c) A sample set generated by Cluster. The blue background is the kernel density estimation result of the samples’ distribution in the dataset. (d) The label distribution under different numbers of samples. (e) The function between the number of samples and the corresponding accuracy.


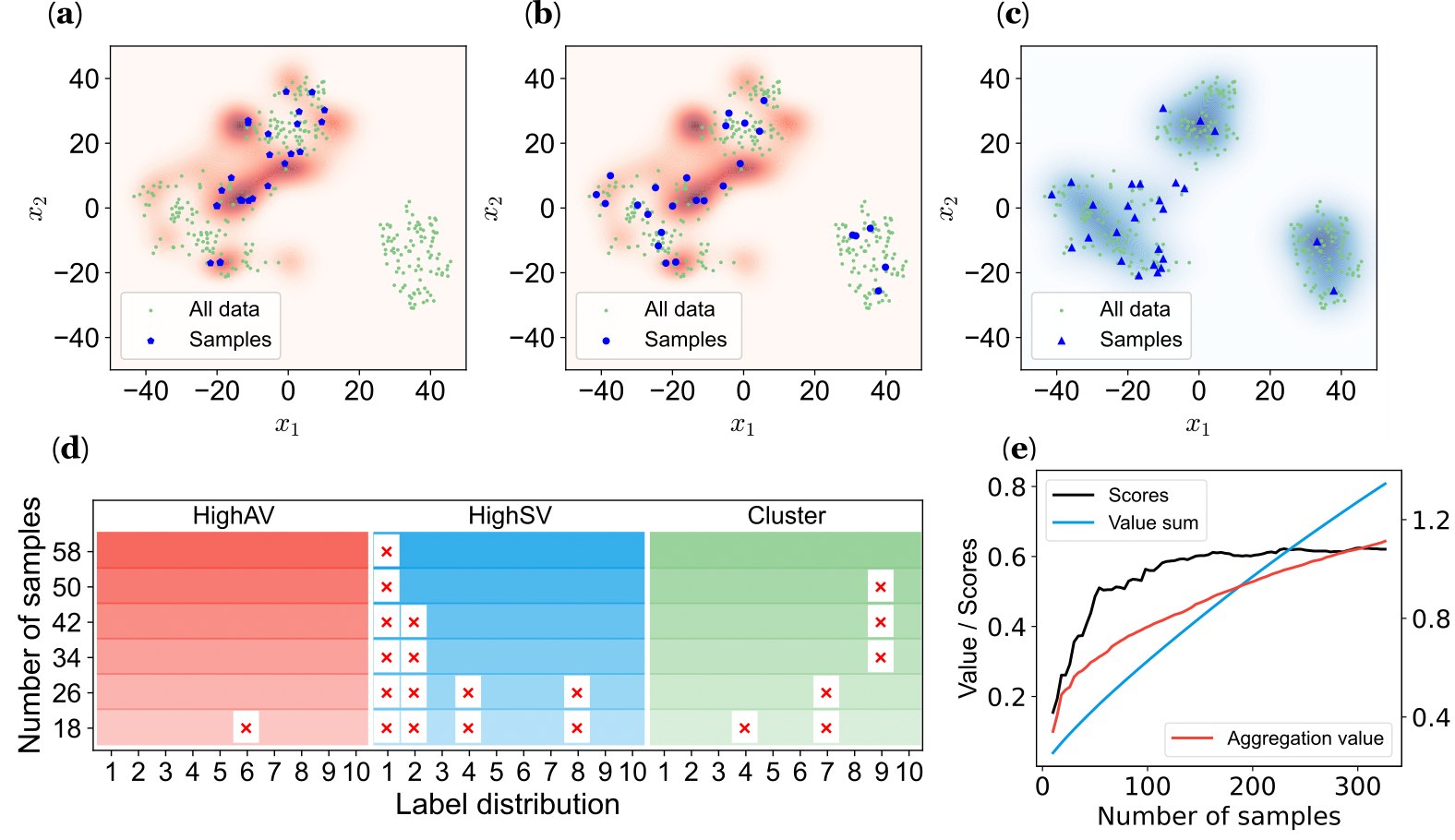


Fig. S 4.2 Characteristics analysis of the CWRU HP1 task. (a) A sample set generated by HighSV. The red background represents the field of Shapley value and the darker colour means a larger value. (b) A sample set generated by HighAV. (c) A sample set generated by Cluster. The blue background is the kernel density estimation result of the samples’ distribution in the dataset. (d) The label distribution under different numbers of samples. (e) The function between the number of samples and the corresponding accuracy.


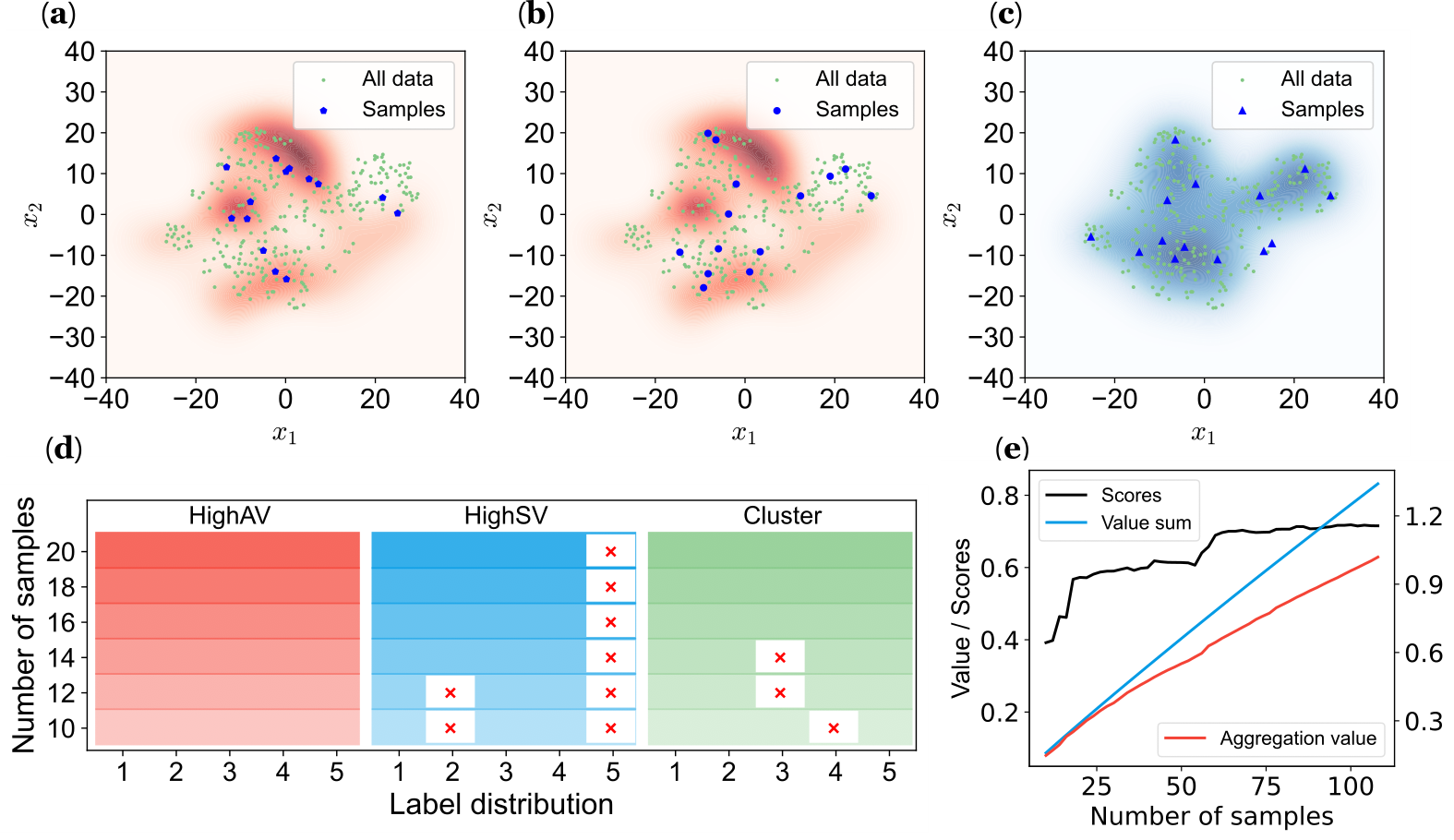


Fig. S 4.3 Characteristics analysis of the Cifar10 task. (a) A sample set generated by HighSV. The red background represents the field of Shapley value and the darker colour means a larger value. (b) A sample set generated by HighAV. (c) A sample set generated by Cluster. The blue background is the kernel density estimation result of the samples’ distribution in the dataset. (d) The label distribution under different numbers of samples. (e) The function between the number of samples and the corresponding accuracy.


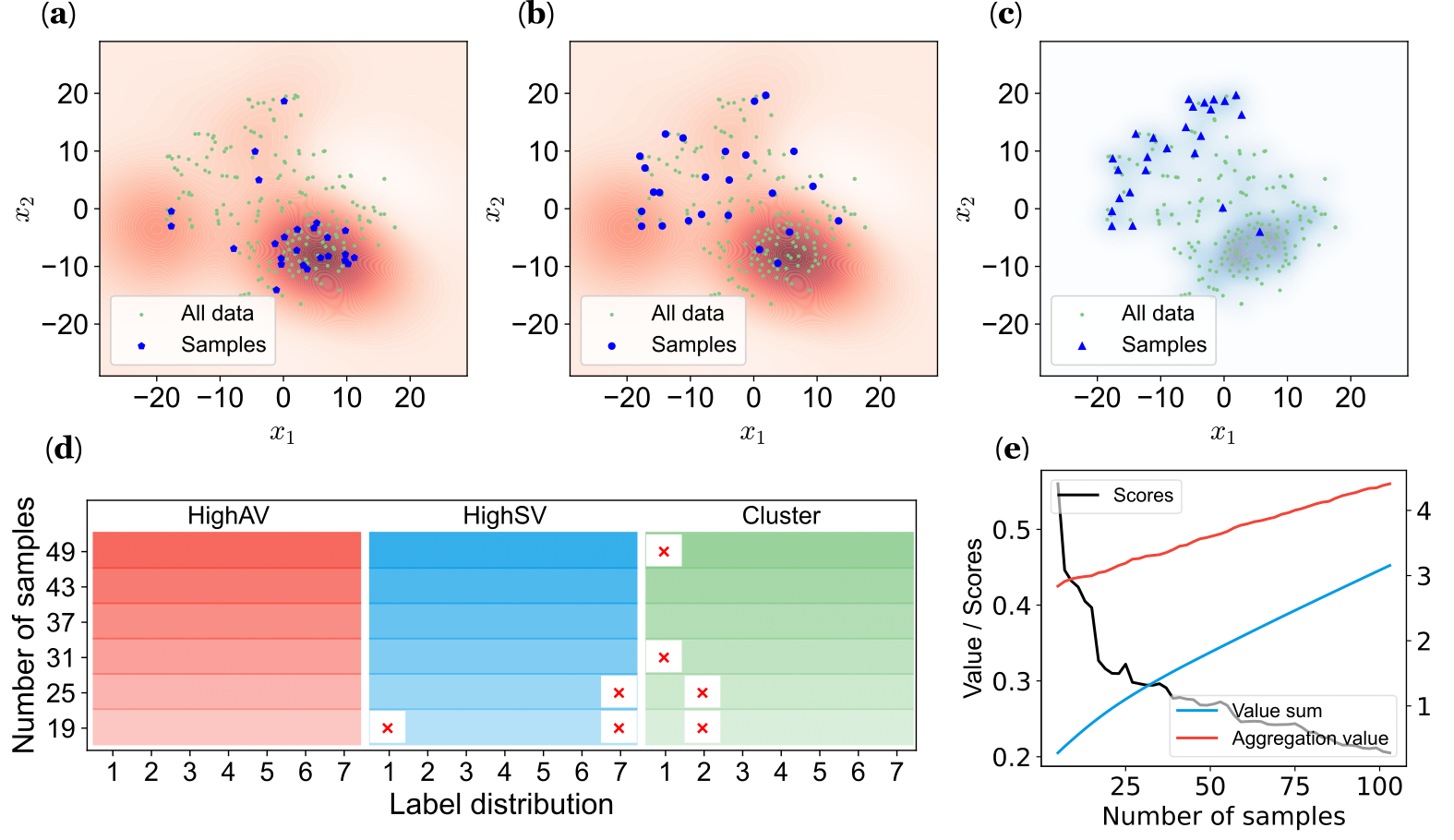


Fig. S 4.4 Characteristics analysis of the Wear B2C4 task. (a) A sample set generated by HighSV. The red background represents the field of Shapley value and the darker colour means a larger value. (b) A sample set generated by HighAV. (c) A sample set generated by Cluster. The blue background is the kernel density estimation result of the samples’ distribution in the dataset. (d) The label distribution under different numbers of samples. (e) The function between the number of samples and the corresponding MAE.


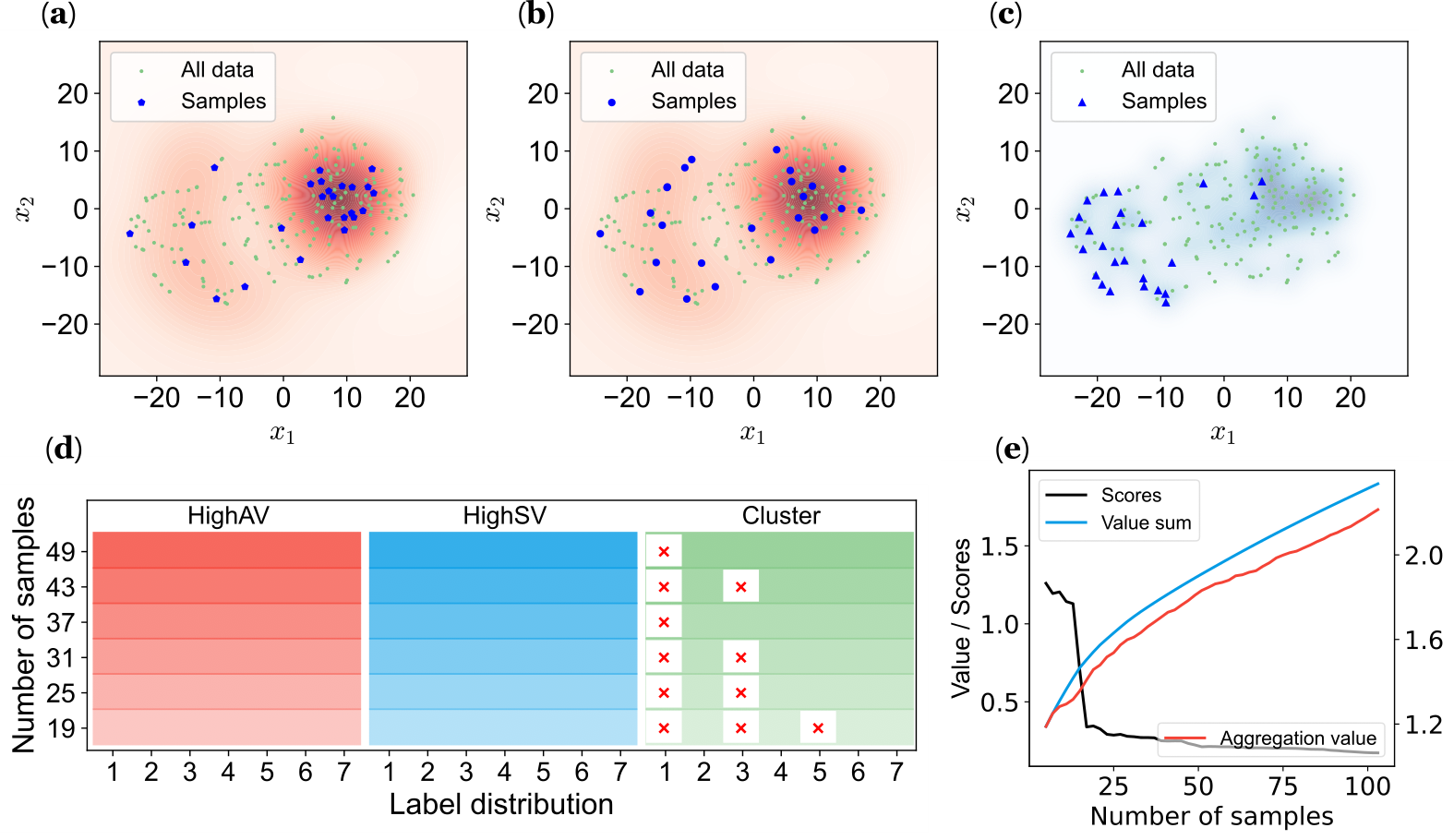


Fig. S 4.5 Characteristics analysis of the Wear B3C6 task. (a) A sample set generated by HighSV. The red background represents the field of Shapley value and the darker colour means a larger value. (b) A sample set generated by HighAV. (c) A sample set generated by Cluster. The blue background is the kernel density estimation result of the samples’ distribution in the dataset. (d) The label distribution under different numbers of samples. (e) The function between the number of samples and the corresponding MAE.

## S4.2 Sensitivity analysis

Since the sensitivity analysis of the composite task was given in the manuscript. This section reports the sensitivity analysis for the rest 5 tasks.


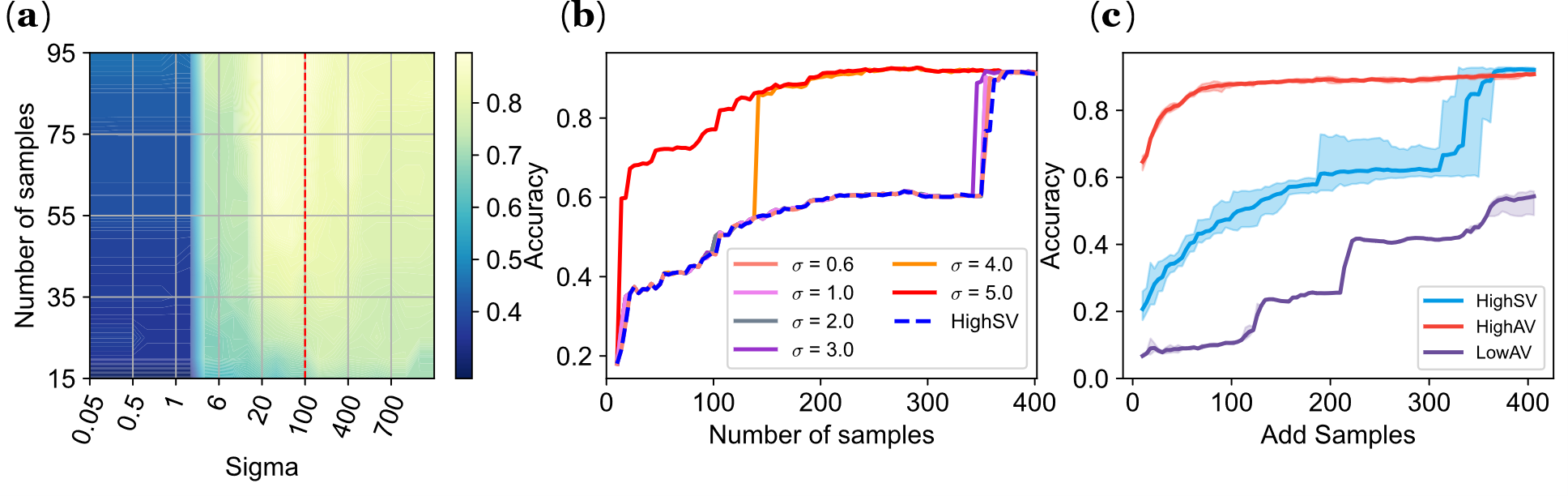


Fig. S 4.6 The sensitivity analysis of the CWRU HP0 task. (a) Sensitivity of parameter kernel width $\boldsymbol{\sigma}$. (b) The degeneration from HighAV to HighSV. (c) Sensitivity on the random error of Shapley value.


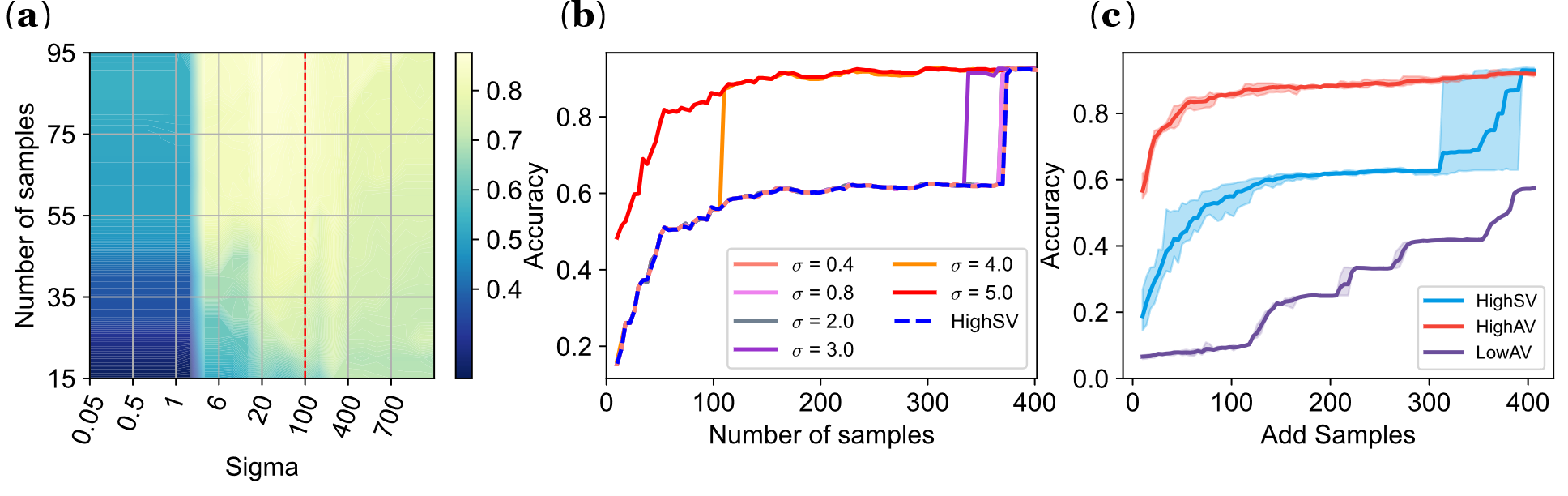


Fig. S 4.7 The sensitivity analysis of the CWRU HP1 task. (a) Sensitivity of parameter kernel width $\boldsymbol{\sigma}$. (b) The degeneration from HighAV to HighSV. (c) Sensitivity on the random error of Shapley value.


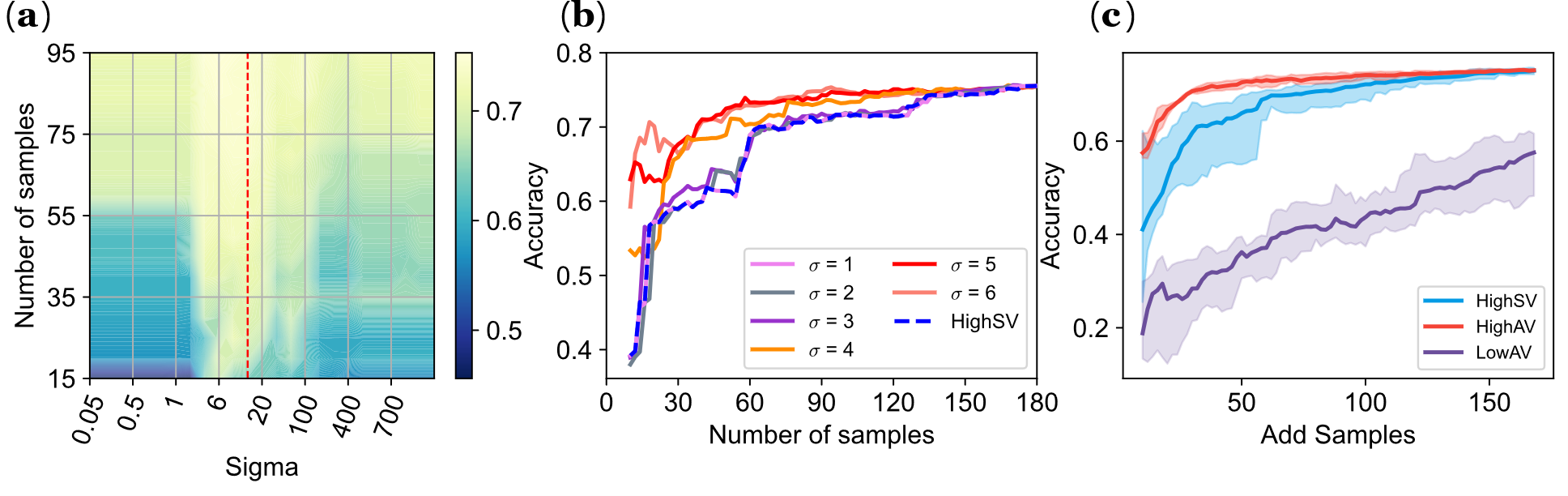


Fig. S 4.8 The sensitivity analysis of the Cifar10 task. (a) Sensitivity of parameter kernel width $\boldsymbol{\sigma}$. (b) The degeneration from HighAV to HighSV. (c) Sensitivity on the random error of Shapley value.


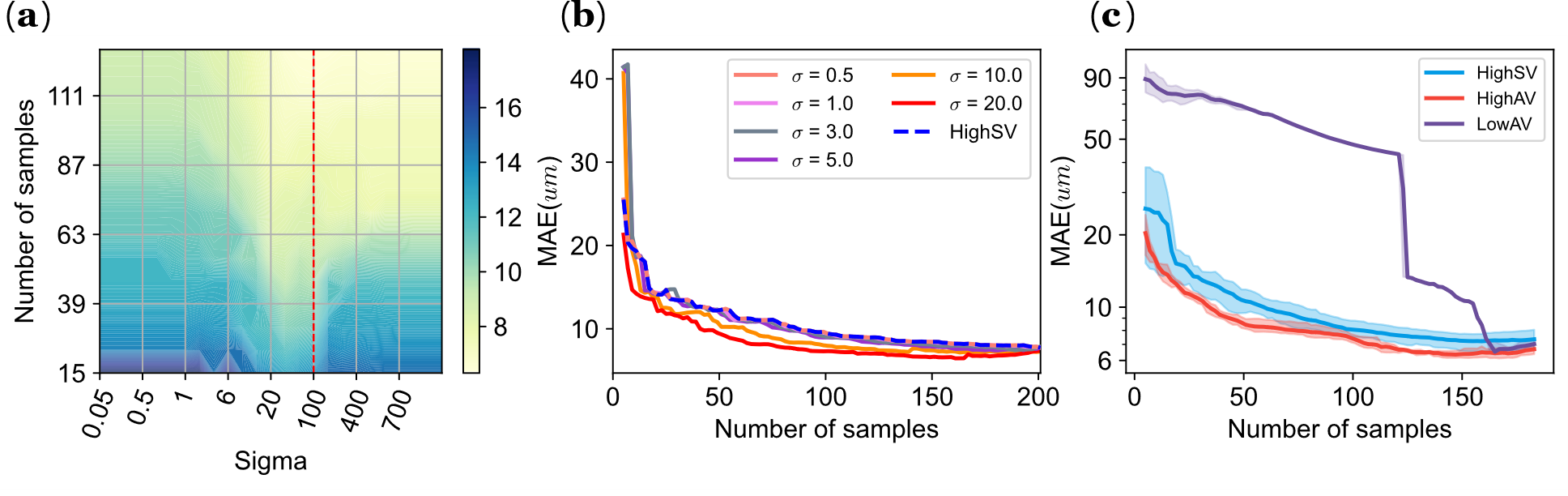


Fig. S 4.9 The sensitivity analysis of the Tool wear B2C4 task. (a) Sensitivity of parameter kernel width $\boldsymbol{\sigma}$. (b) The degeneration from HighAV to HighSV. (c) Sensitivity on the random error of Shapley value.


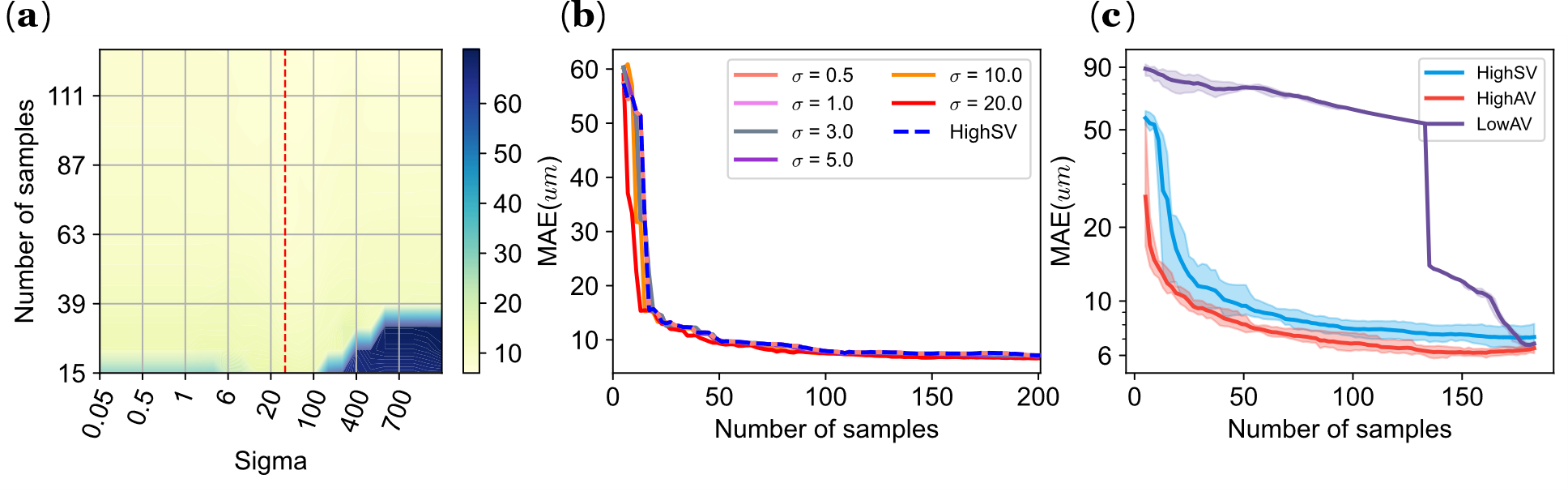


Fig. S 4.10 The sensitivity analysis of the Tool wear B3C6 task. (a) Sensitivity of parameter kernel width $\boldsymbol{\sigma}$. (b) The degeneration from HighAV to HighSV. (c) Sensitivity on the random error of Shapley value.

## S4.3 Comparison of different kernel functions

Fig. S 4.11 shows the performance of the aggregation-value-based sampling (HighAV and LowAV) on the composite task with three different kernel functions, Radial Basis Function (RBF) kernel, Laplace kernel and Inverse Multiquadric (IM) kernel. The upper three curves are results of LowAV, the lowest aggregation value subset. The bottom three curves are the results of HighAV, the highest aggregation value subset. The details of the three kernel functions are listed as follows:

- RBF kernel: $k\left( \boldsymbol{x}_{i},\boldsymbol{x}_{j} \right)=\exp\left( -\frac{\left\| \boldsymbol{x}_{i}-\boldsymbol{x}_{j} \right\|^{2}}{\sigma} \right)$, $\sigma=1e2$. The denominator can also be expressed as the equivalent definition $\sigma=2l^{2}$.
- Laplace kernel: $k\left( \boldsymbol{x}_{i},\boldsymbol{x}_{j} \right)=\exp\left( -\frac{\left\| \boldsymbol{x}_{i}-\boldsymbol{x}_{j} \right\|}{\sigma} \right)$, $\sigma=1e2$.
- IM kernel: $k\left( \boldsymbol{x}_{i},\boldsymbol{x}_{j} \right)=\frac{1}{\sqrt{\left\| \boldsymbol{x}_{i}-\boldsymbol{x}_{j} \right\|^{2}+c^{2}}},c=1$.

*
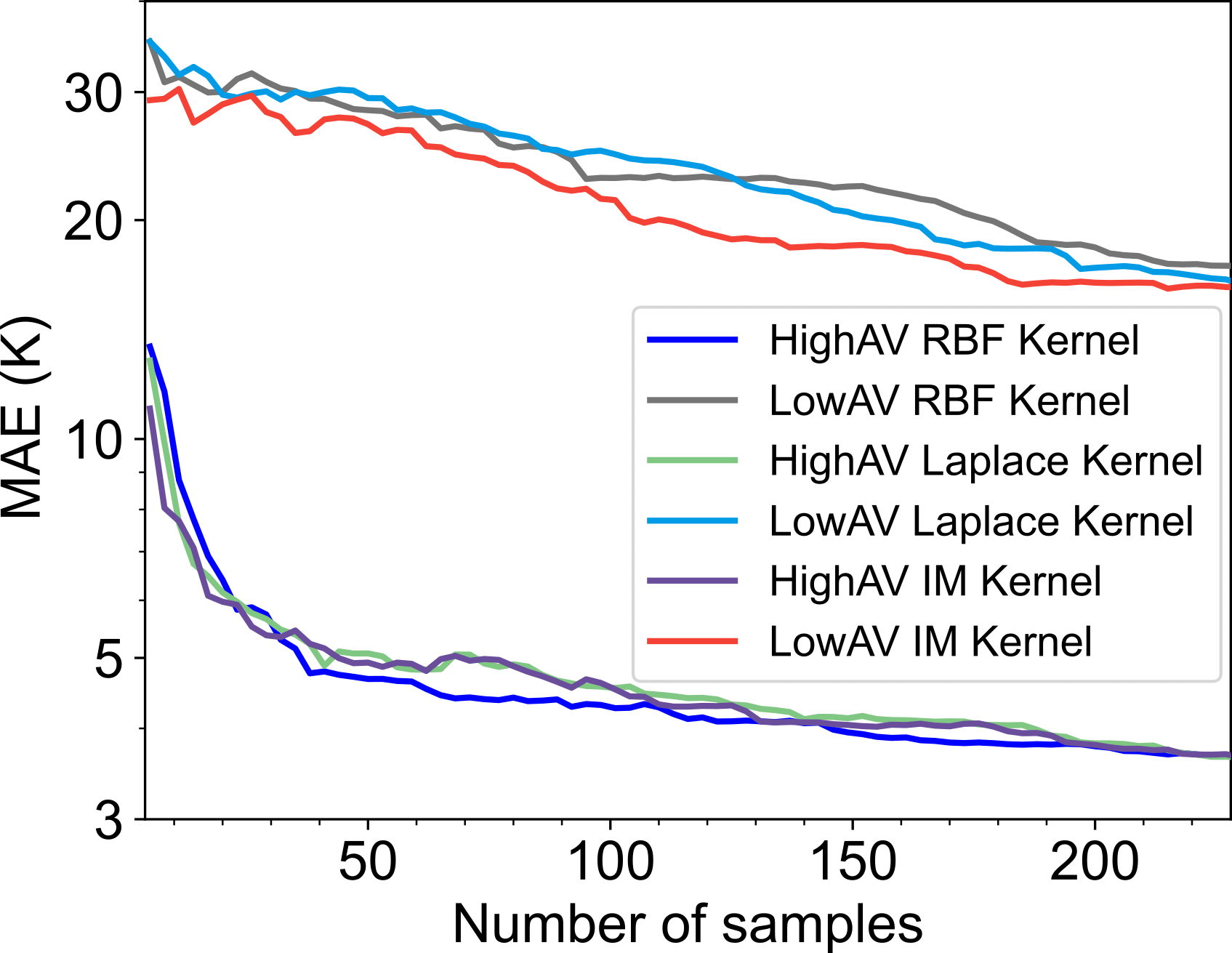
*

Fig. S 4.11 The sensitivity of aggregation-value-based sampling on different kernel functions, Radial Basis Function (RBF) kernel, Laplace kernel and Inverse Multiquadric (IM) kernel.

## S4.4 Discussion about the sub-modularity

**Definition 1 (Marginal gain).** For a set function $\varphi:2^{|N|}\to\mathbb{R}$, a subset $S\subseteq N$, and an element $e\in N$. The marginal gain of $\varphi$ at $S$ respect to $e$ is defined as:

$$\Delta_{\varphi}(e\mid S):=\varphi(S\cup\{e\})-\varphi(S)$$

**Definition 2 (Sub-modularity).** A set function $\varphi:2^{|N|}\to\mathbb{R}$, is submodular if for every $A\subseteq$ $B\subseteq N$, and $e\in N\setminus B$ it holds that:

$$\Delta_{\varphi}(e\mid A)\geq\Delta_{\varphi}(e\mid B)$$

Fig. S 4.12 shows the distribution of the calculated Shapley value for four cases. The title of each figure shows the proportion of points with a negative Shapley value. The Shapley value means the contribution of each sample in the model training, so the outlier or noise may have a negative influence on the modelling, thus resulting in a negative Shapley value. In addition, the uncertainties and approximation error of the modified TMC-Shapley method may also lead to negative Shapley values. Anyway, these negative Shapley value may have two influences on the research problem:

- As shown in **Definition 2**, the sub-modularity requires $\Delta_{\varphi}(e\mid A)\geq\Delta_{\varphi}(e\mid B)$ for $A\subseteq B\subseteq N$ [9]. Suppose set $A$ consists of only a few samples, in contrast, set $B$ has sufficient samples to train a high-accuracy model, namely $\left| A \right|\ll\left| B \right|$. An outlier sample $e$ may reduce the performance of the subset $A$ significantly but only have a few negative influences on the subset $B$, which means $\Delta_{\varphi}(e\mid A)<\Delta_{\varphi}(e\mid B)<0$. Therefore, the aggregation value $\hat{v}_{agg}(S)$ is not a strict sub-modular function anymore.
- The defined aggregation value $\hat{v}_{agg}(S)$ is maximised using the greedy algorithm, but greedy submodular maximisation requires the submodular function to be nonnegative and monotone [9], namely $\hat{v}_{agg}\left( B \right)\geq\hat{v}_{agg}\left( A \right)\geq0$ for any $A\subseteq B\subseteq N$. Therefore, the negative Shapley values will influence the effectiveness of the greedy optimisation.

Although the greedy optimisation can still work for most of situations with negative Shapley value, there is a simple way to avoid the instability. After the calculation of the modified TMC-Shapley method, we subtract the smallest Shapley value in the set from each point $v_{i}$ to keep it nonnegative:$v_{i}= v_{i}-\min\left( v_{i},\cdots,v_{n} \right),$ for $i=1,\cdots,n$*.* After this operation, the function $\hat{v}_{agg}\left( S \right)$ is a strict nonnegative and monotone submodular function, which brings a guarantee for the greedy optimisation of Eq. (10) in the manuscript.

**
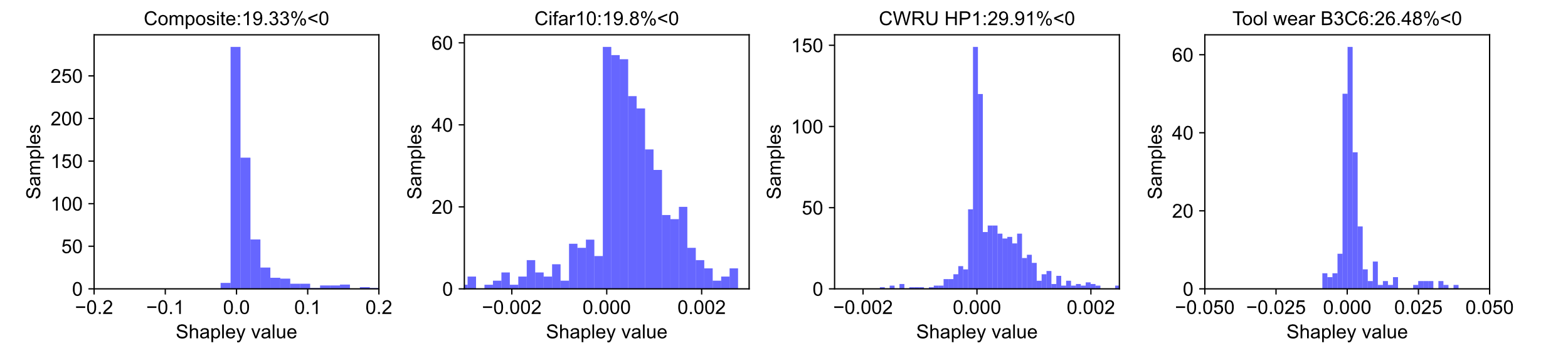
**

Fig. S 4.12 Shapley value distribution for the four cases.

# Reference

1. Ghorbani A and Zou J. Data shapley: Equitable valuation of data for machine learning. In: *Proceedings of the 36th International Conference on Machine Learning (ICML)*. New York, NY: ACM, 2019, 2242–2251.
2. Singh S, Howard CQ and Hansen CH. An extensive review of vibration modelling of rolling element bearings with localised and extended defects. *J Sound Vib* 2015; **357**: 300–30.
3. Bearing Data Center. Case western reserve university seeded fault test. https://engineering.case.edu/bearingdatacenter (10 September 2022, date last accessed).
4. Alex Kriz. The cifar 10 dataset 2022. https://www.cs.toronto.edu/~kriz/cifar.html (10 September 2022, date last accessed).
5. Cuka B, Kim D-W. Fuzzy logic based tool condition monitoring for end-milling. *Robot Comput Integr Manuf* 2017; **47**: 22–36.
6. Rech J, Giovenco A and Courbon C et al. Toward a new tribological approach to predict cutting tool wear. CIRP Annals 2018; **67**: 65–8.
7. PHM society. A phm society conference data challenge, tool wear dataset. https://www.phmsociety.org/competition/phm/10 (10 September 2022, date last accessed).
8. Zobeiry N and Poursartip A. Theory-guided machine learning for process simulation of advanced composites. arXiv:2103.16010.
9. Krause A and Golovin D. Submodular function maximization. *Tractability* 2014; **3**: 71–104.
